# An *N-*glycome tissue atlas of 15 human normal and cancer tissue types determined by MALDI-imaging mass spectrometry

**DOI:** 10.1038/s41598-023-50957-w

**Published:** 2024-01-04

**Authors:** Elizabeth N. Wallace, Connor A. West, Colin T. McDowell, Xiaowei Lu, Evelyn Bruner, Anand S. Mehta, Kiyoko F. Aoki-Kinoshita, Peggi M. Angel, Richard R. Drake

**Affiliations:** 1 Department of Cell and Molecular Pharmacology and Experimental Therapeutics, Medical University of South Carolina, Charleston, SC 29425 USA; 2 Department of Pathology and Laboratory Medicine, Medical University of South Carolina, Charleston, SC 29425 USA; 3 Department of Bioinformatics, Soka University, Tokyo, 192-8577 Japan

**Keywords:** Mass spectrometry, Glycobiology

## Abstract

*N-*glycosylation is an abundant post-translational modification of most cell-surface proteins. *N-*glycans play a crucial role in cellular functions like protein folding, protein localization, cell–cell signaling, and immune detection. As different tissue types display different *N-*glycan profiles, changes in *N-*glycan compositions occur in tissue-specific ways with development of disease, like cancer. However, no comparative atlas resource exists for documenting *N-*glycome alterations across various human tissue types, particularly comparing normal and cancerous tissues. In order to study a broad range of human tissue *N*-glycomes, *N*-glycan targeted MALDI imaging mass spectrometry was applied to custom formalin-fixed paraffin-embedded tissue microarrays. These encompassed fifteen human tissue types including bladder, breast, cervix, colon, esophagus, gastric, kidney, liver, lung, pancreas, prostate, sarcoma, skin, thyroid, and uterus. Each array contained both normal and tumor cores from the same pathology block, selected by a pathologist, allowing more in-depth comparisons of the *N-*glycome differences between tumor and normal and across tissue types. Using established MALDI-IMS workflows and existing *N-*glycan databases, the *N-*glycans present in each tissue core were spatially profiled and peak intensity data compiled for comparative analyses. Further structural information was determined for core fucosylation using endoglycosidase F3, and differentiation of sialic acid linkages through stabilization chemistry. Glycan structural differences across the tissue types were compared for oligomannose levels, branching complexity, presence of bisecting *N-*acetylglucosamine, fucosylation, and sialylation. Collectively, our research identified the *N-*glycans that were significantly increased and/or decreased in relative abundance in cancer for each tissue type. This study offers valuable information on a wide scale for both normal and cancerous tissues, serving as a reference for future studies and potential diagnostic applications of MALDI-IMS.

## Introduction

Determining the molecular composition of cells and tissues using “omic” technologies provides key research insights on pathway and cellular behavior in normal tissues, cancer, and other diseases. These approaches serve as a window into what is normal and what pathways, mechanisms, and interactions become dysregulated in disease states. Currently the Human Genome Project and the Human Proteome Project are two of the largest and most established of such omics programs. The Human Genome Project (https://www.genome.gov/human-genome-project), begun in 1988, has successfully sequenced the entire human genome assembled from the genetics of numerous volunteers, providing extremely valuable information on codes for proteins, non-coding RNA, and gene regulation, as well as leading to the emergence of proteomics^1,2^. This has led to the still-ongoing Human Proteome Project (https://www.hupo.org/human-proteome-project/), which seeks to identify all the proteins coded for in human cells and biofluids, with the ability to study effects of gene knockouts or overexpression, post-translational modifications (PTMs), and protein–protein interactions^3^. Two large atlasing efforts for the human proteome and cellular data include the Human Protein Atlas^4^ (https://www.proteinatlas.org) and the Human Cell Atlas^5^ (https://www.humancellatlas.org). There is also an extensive resource for metabolites accessible in the Human Metabolite Database (https://www.hmdb.ca) as part of the Human Metabolome Project ^6^. The information assembled in these databases provide big data resources that can inform human biomedical studies of varying scale.

Information on the human glycome, the repertoire of simple and complex carbohydrates present inside cells, on the cell surfaces as part of their glycocalyx, and in secretomes, is much more limited relative to the aforementioned atlas and omic projects. A challenge in studying glycans and glycosylation on an omics level comes from the fact that glycan structures are not template-driven by the genome. The pioneering Human Glycome Project initiative has been created, with initial emphasis on comprehensive characterization of glycosylation of immunoglobulins and other blood glycoproteins^7^. Defining the glycan constituents of serum and plasma glycoproteins could be applied to many biomedical applications, including diagnostic biomarkers^7–10^; however, the link between biofluid and tissue glycosylation is not well established and biofluid data lacks the critical spatial expression features identified in tissue analyses^9^. Systematic evaluation of tissue glycosylation will lead to improved understanding of the human glycome in its entirety and create a database of tissue glycosylation to supplement and expand what is already known^11^.

Beyond what is encoded by the transcriptome for protein translation, a multitude of different post-translational modifications (PTMs) are done in the cell that affect signal transduction, protein folding, protein localization, and protein complex formations. One of the most diverse and structurally complex PTMs is glycosylation, the addition of carbohydrates to proteins or lipids ranging from single monosaccharides to complex carbohydrates in branched or polymeric chains^12,13^. A major class of glycan PTMs are the asparagine-linked *N-*glycans, which function as key components of many cellular functions like protein folding, vesicular transport, immune cell interactions, and cell–cell recognition processes^12–18^. In the realm of diseases, especially cancer, structural changes to *N-*glycans have been studied as both drivers of and biomarkers for disease development and progression^10,11,19–21^. Biosynthetically, *N-*glycans arise from the transfer of dolichol-linked oligosaccharide precursors co-translationally to proteins in the endoplasmic reticulum at consensus sites of Asn-X-Ser/Thr. A series of glycosidases and glycosyltransferases processively modify attached *N-*glycans in the ER and Golgi. This complex pathway of formation involves not only various cellular locations but also a wide network of hundreds of genes involved in biosynthesis and processing^13,22^.

Within the domain of *N*-glycans, a profound layer of complexity emerges from their intricate structural composition. These intricate glycoconjugates are assembled from combinations of monosaccharides and modifiers such as sulfates. The most common monosaccharides seen in *N-*glycans are mannose, galactose, *N-*acetylglucosamine, sialic acid/*N-*acetylneuraminic acid, *N-*acetylgalactosamine, fucose, or glucose. It is estimated that there are at least 2000 possible *N-*glycan compositions^12^, and these arise from different combinations of the primary monosaccharide species, anomeric and linkage differences, branching/extensions, and other modifications such as sulfate or phosphate additions^14^. To add further intricacy, the location and anomeric configurations of individual sugars can radically alter glycoprotein function and localization. Because of these cumulative challenges, no overview of normal tissue and cancerous *N-*glycomes currently exists, as most studies have focused on characterizing single tissue and cancer types.

Specific structural *N-*glycan motifs have been associated with normal and pathological functions. High-mannose *N-*glycans are often elevated in fast-growing cancers, and generally correlate to increased metastasis in those cancers^23–26^. Conversely, *N-*glycans with a bisecting *N-*acetylglucosamine (GlcNAc) residue can either drive or inhibit cancer growth depending on which proteins they modify^27–29^. Fucose-containing *N-*glycans, particularly those with core fucosylation, have been associated by clinical analysis and experimentation with more dangerous cancer phenotypes, metastasis, and poor survival outcomes^26,30–32^. Additionally, sialic acid carbohydrate tumor antigens, termed sialyl Lewis X and sialyl Lewis A (also called CA19-9), and other sialylated glycans have been evaluated in many cancer types for decades^33–36^. To better inform the trajectory of future studies focused on cancer development, progression, and targeted tissue-specific treatment responses, a comprehensive mapping of glycan structural trends within healthy tissues and their transformations in the context of cancer is imperative. This holistic understanding will unveil critical insights and better inform future studies of cancer development, progression, and treatment responses in targeted tissues.

Mass spectrometry-based analysis of *N-*glycans has emerged in the last 20 years as the most effective way to evaluate the diverse structural and chemical properties of *N-*glycans in biological samples^11,37–39^. Imaging mass spectrometry (IMS) applications to biological tissues have proven to be an effective approach to identify the structural compositions, tissue distribution, and localization of *N-*glycans^11,40^. The overall methodological workflow for *N-*glycan IMS has continued to evolve in concert with continued improvements in enzymes utilized, instrumentation, software, and multimodal spatial-omics technologies^40,41^. The method is ideal for mapping the human tissue *N-*glycome, as it uses donor samples directly from pathology archives, allowing expert annotation and selection of tissue features to distinguish normal from disease histologies. Hence, *N-*glycans can be detected by mass spectrometry and mapped to specific locations in tissues to inform what *N-*glycan species are present and their relative abundance for comparisons with other samples^11^.

This study has developed a human tissue glycome resource for *N-*glycosylation, analyzing the *N-*glycome profiles across fifteen major tissue types with paired normal and tumor tissues selected by a pathologist from archival collections. Using a tissue microarray (TMA) format and representative full slide formalin-fixed paraffin-embedded (FFPE) tissues, the relative abundances of individual *N-*glycan compositions were determined by *N-*glycan IMS, assessing changes between normal and tumor glycan relative abundance, what types of *N-*glycans are most abundant in each tissue type, and what *N-*glycan changes occur in the different types of cancers. In addition to using PNGaseF to release the tissue *N-*glycans, the relative abundance of core fucosylation and sialic acid isomers are also evaluated using isomer specific enzymes^42^ and chemical stabilization strategies^43^. Using a TMA for our cohort increases the ability to profile on such a scale and with such variety, allowing for broader information and more in-depth analysis. Such an overview of important tissue types will give structural and biological information on a wide scale, as well as provide reference points for diagnostics and highlight key cancer-related *N-*glycans by tissue to inform further research. This is the first comprehensive glycoTMA that builds on previous findings in the field and shows a broad range of tissues with comparisons not only between tissues and their respective cancers but between tissue types as well. With the increase in MALDI-IMS across this field, this study demonstrates the broad application of these techniques in profiling, showcases a broad TMA useful for profiling of many sorts, and provides a valuable reference for future research on any of these tissues. This data will be available on METASPACE, an open-access imzML database for imaging mass spectrometry data^44,45^, and GlyCosmos^46^, allowing these results to serve as the groundwork for future studies.

## Results

### *N-*glycan profiling with PNGase in custom tissue microarrays

**Figure 1:**
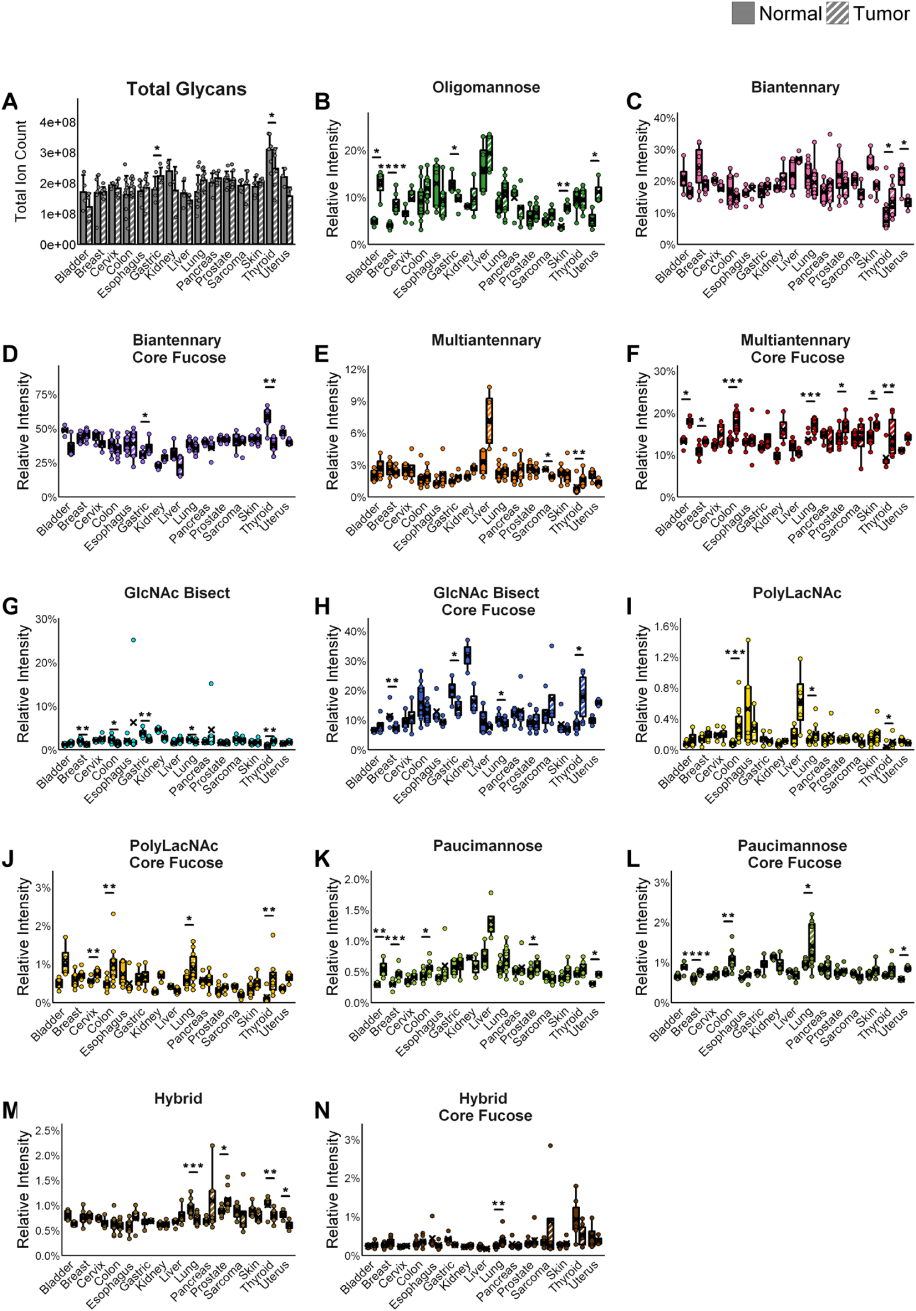
*N-*glycan distributions by glycan type for each tissue (bladder n = 4, breast n = 7, cervix n = 4, colon n = 10, esophagus n = 5, gastric n = 4, kidney n = 3, liver n = 4, lung n = 10, sarcoma n = 4, skin n = 5, pancreas n = 5, prostate n = 8, thyroid n = 6, uterus n = 3). Significance is marked as follows: (*): p-value < 0.05; (**): p-value < 0.01; (***): p-value < 0.001; (****): p-value < 0.0001. Error bars on bar charts represent one standard deviation. Error bars on boxplots represent the quartiles. (**A**) Total *N*-glycan abundance (total ion count) by tissue type. (**B–N**) *N*-glycans graphed by *N*-glycan category. (**B**) Oligomannose *N*-glycans. (**C**) Biantennary *N-*glycans—no core fucose. (**D**) Biantennary *N-*glycans—core fucose. (**E**) Multiantennary *N-*glycans—no core fucose. (**F**) Multiantennary *N*-glycans—core fucose. (**G**) GlcNAc bisect *N*-glycans—no core fucose. (**H**) GlcNAc bisect *N*-glycans—core fucose. (**I**) PolyLacNAc *N*-glycans—no core fucose. (**J**) PolyLacNAc *N*-glycans—core fucose. (**K**) Paucimannose *N*-glycans—no core fucose. (**L**) Paucimannose *N-*glycans—core fucose. (**M**) Hybrid *N*-glycans—no core fucose. (**N**) Hybrid *N*-glycans—core fucose.

Two custom FFPE TMAs representing 89 tumor and normal core pairs from fifteen human tissue types were processed for *N-*glycan imaging mass spectrometry on a MALDI-FTICR mass spectrometer as described in Materials and Methods (Supplementary Table 1). After assigning glycan structural compositions using established *N-*glycan MALDI databases^34^,and using the corresponding peak intensities for each glycan in each tissue core, a unified peak list of 97 *N-*glycan compositions and peak intensity data set was selected (Supplementary Table 2). This peak intensity data was used to assess relative intensities of individual *N-*glycan species across all tissue types to determine proportionate relative abundances in tumor and normal conditions (Fig. 1). Of the 15 pairs, the tissue with the highest overall *N-*glycan relative abundance was the normal thyroid tissue, which was also significantly higher than the corresponding thyroid cancer tissue (Fig. 1A). This is likely due to the fact that thyroglobulin, the backbone for hormone synthesis in the thyroid, requires *N-*glycosylation to function, as do thyroid stimulating hormone and important receptors in the thyroid, activity likely lost in cancer^47–49^. Bladder tissue had the lowest overall intensities, with liver as second lowest. For reference, the top 20 *N-*glycans with the highest intensities in all tissue cores are listed in Supplementary Fig. 1. A simple clustering comparison was done for each tissue group and the 97 *N-*glycans detected (Supplementary Fig. 2). For normal tissues, the thyroid was most unique relative to the other tissues, and broadly, gastrointestinal normal tissues clustered together. Liver cancer tissues were the most distinct among the tumor group (Supplementary Fig. 2).

The glycan at 1809.64 *m/z*, Hex5dHex1HexNAc4, is the most highly detected in nearly all tissues, closely followed in most tissues by 1663.58 *m/z*, Hex5HexNAc4, which has the same structure but lacks a core fucose. This glycan structure is a simple biantennary *N-*glycan that is the natural endpoint of the basic *N-*glycan biosynthetic pathway, with or without core fucosylation^14,50^. The triantennary (*m/z* = 2174.77), bisected (*m/z* = 2012.72), and sialylated (*m/z* = 2122.73) forms of 1809.64 are also among the most abundant glycans seen overall, and the majority of the top 20 glycans detected overall are either fucosylated, sialylated, bisected, and/or further branched versions of these top two glycans (Supplementary Fig. 1). Four examples of glycan intensity images from this list are shown in Supplementary Fig. 3 for both TMAs. The remainder of the top glycans are the five main high-mannose glycans, which form an earlier part of the glycan biosynthetic pathway^14,50^ (Supplementary Fig. 1).

**Figure 2:**
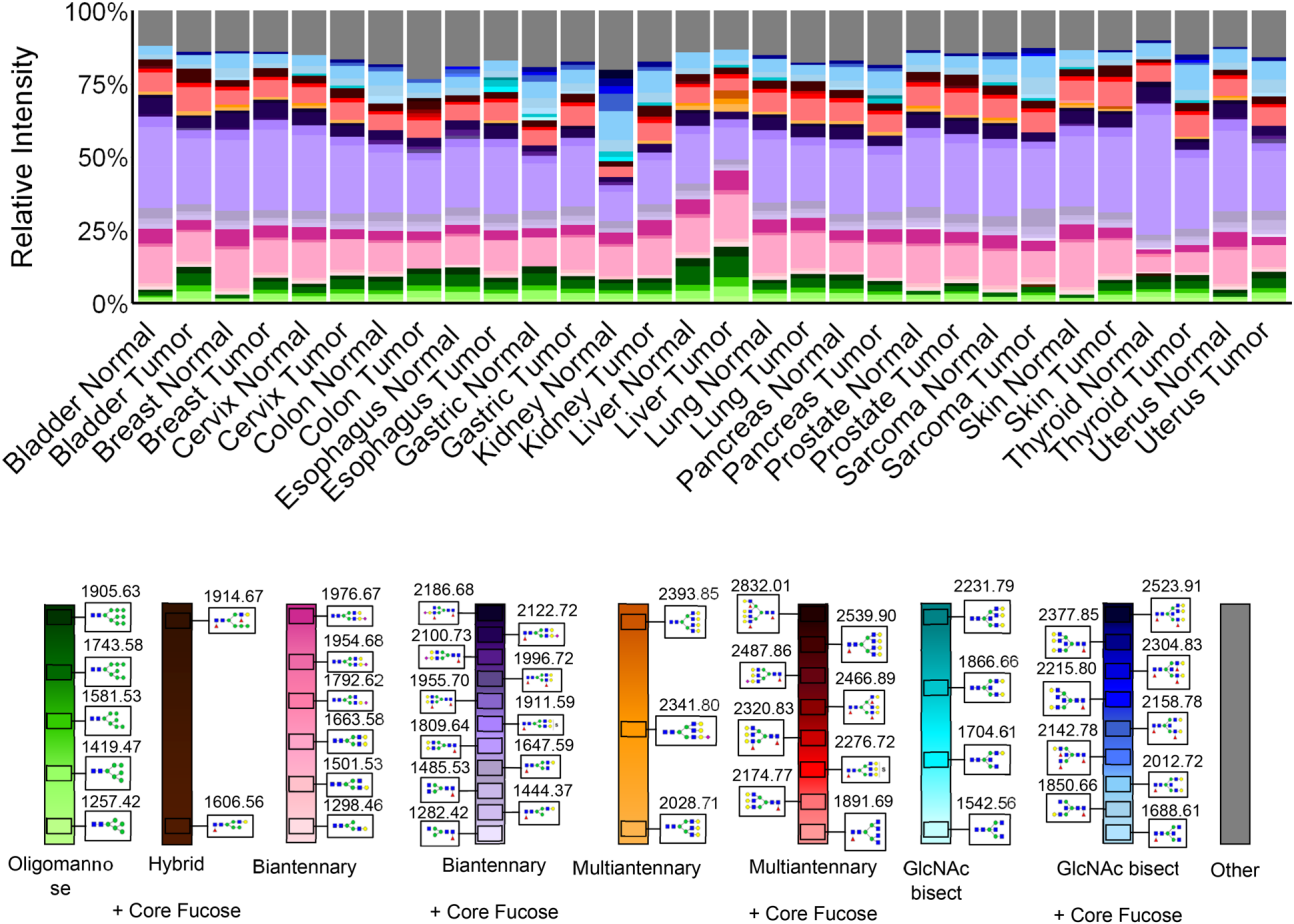
Top 25 *N-*glycans per tissue. Average relative intensities of top 25 *N-*glycans for each tissue type sorted by *N-*glycan type (bladder n = 4, breast n = 7, cervix n = 4, colon n = 10, esophagus n = 5, gastric n = 4, kidney n = 3, liver n = 4, lung n = 10, sarcoma n = 4, skin n = 5, pancreas n = 5, prostate n = 8, thyroid n = 6, uterus n = 3). Each column shows the top 25 *N-*glycans for that tissue, with the grey portion representing the sum of remaining *N-*glycans. The legend shows glycan structures and glycans are arranged by glycan type in a gradient from smallest *m/z* to largest.

Next, the 97 *N-*glycans were segregated into seven broad structural classes: oligomannose, biantennary, multiantennary, GlcNAc bisect, polylactosamine (polyLacNAc), paucimannose, and hybrid, then further separated by presence or absence of core fucose. For these groupings, the presence of sialic acids was also included for each structural class for data in Fig. 1B–N. Comparisons of the total peak intensities for all normal and tumor cores for each of the seven groups are graphed in Supplementary Fig. 4. The average relative intensities of all *N-*glycans detected for each tissue type were compiled and used to determine the top 25 *N-*glycans detected in each tissue type, shown graphically in Fig. 2.

### Oligomannose *N-*glycans

For the oligomannose category, the mean percentage across all tissues was 9%, ranging from 3 to 23%. Notably, in a majority of cancer types, there is a higher relative abundance of the oligomannose *N-*glycans as compared to normal tissue. In Supplementary Fig. 5, the intensities of the main five oligomannose species, i.e., Man5–Man9, are shown for each tumor type. Overall, the most abundant detected oligomannose species is Man8, at *m/z* = 1743.58. These *N-*glycans are associated with rapid cell growth and, in many cases, have been found to correlate to cancer growth^23–26,51–54^. Bladder, breast, and skin all show significantly elevated levels of oligomannose *N-*glycans in cancer, with the breast showing the most significant difference (p < 0.001) (Fig. 1B, Supplementary Figs. 5–7, 18). Individual oligomannose *N-*glycans show significance with higher relative abundance in cancer in bladder, breast, colon, kidney, liver, skin, and uterus (Supplementary Figs. 5–7, 9, 12–13, 18, 20).

However, it is striking to note that in several cancer types, oligomannose *N-*glycan relative abundance was lower in cancer than healthy tissue—significantly so in the case of gastric cancer, with the same trend in pancreas, though only when the overall category is compared, as individual oligomannose *N*-glycans reflect the trend but no significance (Fig. 1B, Supplementary Figs. 5, 11, 15). Individual oligomannose *N-*glycans show significance with lower relative abundance in cancer in cervix, esophagus, and uterus (Supplementary Figs. 5, 8, 10, 20). This is in contrast to what is generally expected for oligomannose *N-*glycans in cancer and highlights the importance of knowing glycan relative abundance trends in different tissue types as well as how these change in cancer for each tissue type.

### Biantennary and multiantennary *N-*glycans

The most abundant structural class detected were the biantennary *N*-glycans, with a mean percentage across all tissues for non-core-fucosylated of 19%, ranging from 5 to 35% and for core-fucosylated of 39%, ranging from 15 to 67%. These *N-*glycans were usually present at higher levels in healthy tissues as compared to cancer tissues, with uterus showing significance for core-fucosylated and thyroid showing significance in non-core-fucosylated. In contrast, gastric tissue showed significantly higher core-fucosylated biantennary *N-*glycan relative abundance in cancer tissue than normal, as did thyroid in non-core-fucosylated (Fig. 1C,D, Supplementary Figs. 11, 19–20). Most tissues also show significance in individual biantennary *N-*glycans (Supplementary Figs. 6–7, 9–10, 12–14, 16–20).

Multiantennary *N-*glycans, which include both tri- and tetra-antennary structures, were detected with a mean percentage across all tissues of 2%, ranging from 0.4 to 10% for non-core fucosylated and a mean of 14%, ranging from 7 to 22% for core-fucosylated. Highly branched *N-*glycans, and particularly those with core fucose, have been associated with cancer progression both as drivers and products of dysregulation^26,30,31,55^. When core-fucosylated, these large *N-*glycans were increased in most of the cancer types, with significantly higher relative abundance (p < 0.05) in bladder, breast, colon, lung, prostate, skin, and thyroid cancers (Fig. 1F, Supplementary Figs. 6–7, 9, 14, 16, 18–19). The highly branched *N-*glycans with no fucosylation are seen to be significantly more abundant in thyroid cancer compared to normal tissue, and less abundant in sarcoma compared to the corresponding normal tissue (Fig. 1E, Supplementary Figs. 17, 20). These are also overall highest in liver cancer and among the top *N-*glycans for these samples, though not statistically significant compared to normal liver tissue due to limited sample size and wide variation between samples (Figs. 1E, 2, Supplementary Fig. 13). This result supports the established research showing increased *N-*glycan branching with and without fucosylation in hepatocellular carcinoma, as well as increases in non-fucosylated highly branched *N-*glycans linked with genetic subtyping^31,55^. These *N-*glycans are also present in the top 25 for normal liver tissue but not seen in the top 25 *N-*glycans for most other tissue types. These *N-*glycans have a role in signaling pathways in healthy livers, being linked to upregulated fatty acid uptake, although the specifics of what role they play is unclear^56^. Almost all tissues also show significance between healthy tissue and cancer in individual multiantennary *N-*glycans (Supplementary Figs. 6–12, 14–20).

### Bisecting *N-*acetylglucosamine *N-*glycans

*N-*glycans containing a bisecting *N-*acetylglucosamine (GlcNAc) were detected with a mean percentage across all tissues of 2%, ranging from 0.6 to 25% for non-core fucosylated and a mean of 12%, ranging from 5 to 37% for core-fucosylated. These *N-*glycans varied widely in whether they showed higher or lower relative abundance in cancer, with overall significance both with and without a core fucosylation in breast, gastric, lung and thyroid tissues, and significance only without a core fucosylation in colon tissue (Fig. 1G, H, Supplementary Figs. 7, 9, 11, 14, 19). These *N-*glycans are involved in cell–cell signaling and also commonly associated with IgGs and immune invasion, factors which also vary among cancers^27,28^. As expected^57^, kidney tissues showed particularly high levels of bisecting GlcNAc *N-*glycans in normal tissue, though not statistically significant from its cancer, likely due to low sample size and wide variation between tissues (Fig. 1G, H and Supplementary Fig. 12). GlcNAc-bisected *N-*glycans are known to play key regulatory roles in the kidneys and be most highly detected in kidney and neural tissue^27,29,57^. Because these *N-*glycans are tubule-associated, the GlcNAc bisects are much less abundant in the tumors. This reflects an overall trend observed in both the top 25 *N-*glycans and the total core fucosylated *N-*glycans, where when one type of *N-*glycan formed a sizable percentage of the *N-*glycan distribution in normal tissue, it was less present in cancer and vice versa. Individual GlcNAc bisects show significance in most tissues, with the most individually significant of these in the kidney (Supplementary Figs. 6–9, 11–14, 16–20).

### Polylactosamine, hybrid and paucimannose *N-*glycans

Another class of *N-*glycan assessed for significance was *N-*glycans with polyLacNAc extensions, a type of *N-*glycan containing a branch terminus repeat of galactose and *N-*acetylglucosamine dimers. PolyLacNAc glycans were detected with a mean percentage across all tissues of 0.2%, ranging from 0.007 to 1.4%, for non-core fucosylated and a mean of 0.6%, ranging from 0.03 to 2%, for core-fucosylated. This *N-*glycan type was significantly more abundant compared to normal tissue in colon, lung, and thyroid cancers with and without core fucosylation, and in cervix when core fucosylated (Fig. 1I–J, Supplementary Figs. 8–9, 14, 19). Although low in overall relative abundance, PolyLacNAc *N-*glycans have been previously associated with increased aggressiveness and metastasis in various cancers^58,59^, and breast cancer in particular^30,32^. Paucimannose and hybrid *N-*glycan showed overall very low relative abundances (0.1%-3%), but the overall relative abundances and significance followed the trends of oligomannose and multiantennary, respectively (Fig. 1G, H).

### Other structural comparisons

It was observed that the total amount of *N-*glycans, that is, the total ion count numbers before relative abundance normalization, may increase or decrease from normal to tumor (Fig. 1A), and this appears to be tissue-specific. The changes for individual glycan species within each tumor tissue compared to normal was also evaluated for statistical significance (Supplementary Figs. S6–S20). Of these, the most commonly elevated in cancer—with significantly higher relative abundance in five or more tissues—are the *N-*glycans seen in Table 1.

**Table 1:** *N-*glycans with significantly higher relative abundance in cancer in > 5 tissues.

*m/z*	Name	# tissues significant	# tissues elevated in cancer	*N-*glycan type
771.2822	Hex2HexNAc2	6	6	Paucimannose
2539.90	Hex7dHex1HexNAc6	6	6	Multiantennary
3635.30	Hex10dHex1HexNAc9	6	5	PolyLacNAc
1079.37	Hex3dHex1HexNAc2	5	5	Paucimannose

Two of these four *N-*glycans, one of the two *N-*glycans most often increased in cancer at 771.28 *m/z* and the *N-*glycan at 1079.37 *m/z* are paucimannose, possibly indicating truncation of the *N-*glycans or increased signaling for protein degradation^60–62^. The *N-*glycan at 3635.30 *m/z* is interesting as it is significant in six of the fifteen tissues and seen in higher relative abundance in cancer in five, and it is the only polyLacNAc glycan in this list. The remaining *N-*glycan in this list, the second *N-*glycan elevated in cancer in all instances where it is significant, is multiantennary, which fits with the known trends of multiantennary *N-*glycans being often elevated in cancer^26,30,31,51–55^.

### Full tissue *N-*glycan imaging mass spectrometry

**Figure 3:**
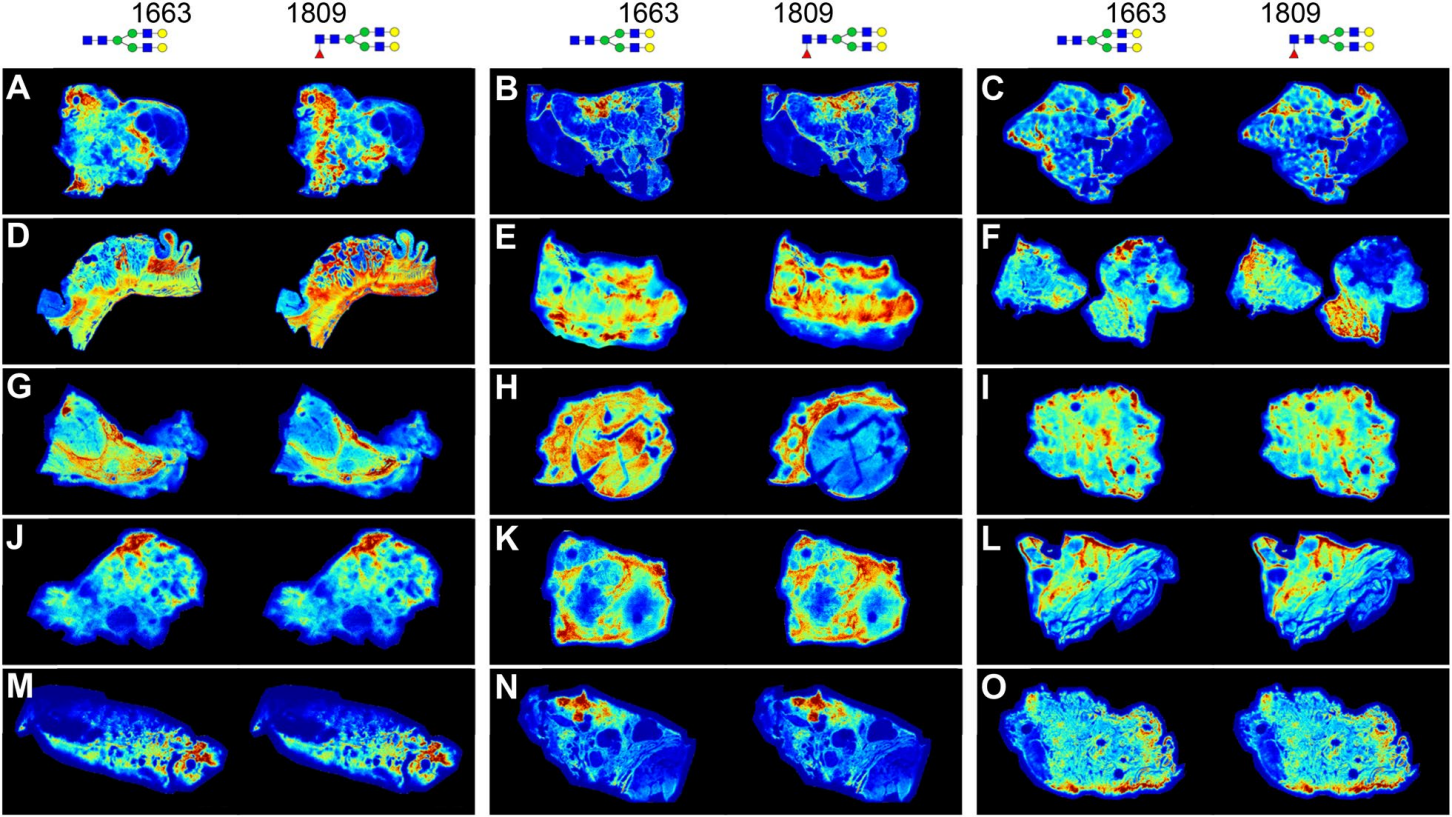
Representative tissues from each tissue type showing *N-*glycans at *m/z* 1663 and 1809. (**A**) Bladder, (**B**) breast, (**C**) cervix, (**D**) colon, (**E**) esophagus, (**F**) gastric, (**G**) kidney, (**H**) liver, (**I**) lung, (**J**) pancreas, (**K**) prostate, (**L**) sarcoma, (**M**) skin, (**N**) thyroid, (**O**) uterus.

**Figure 4:**
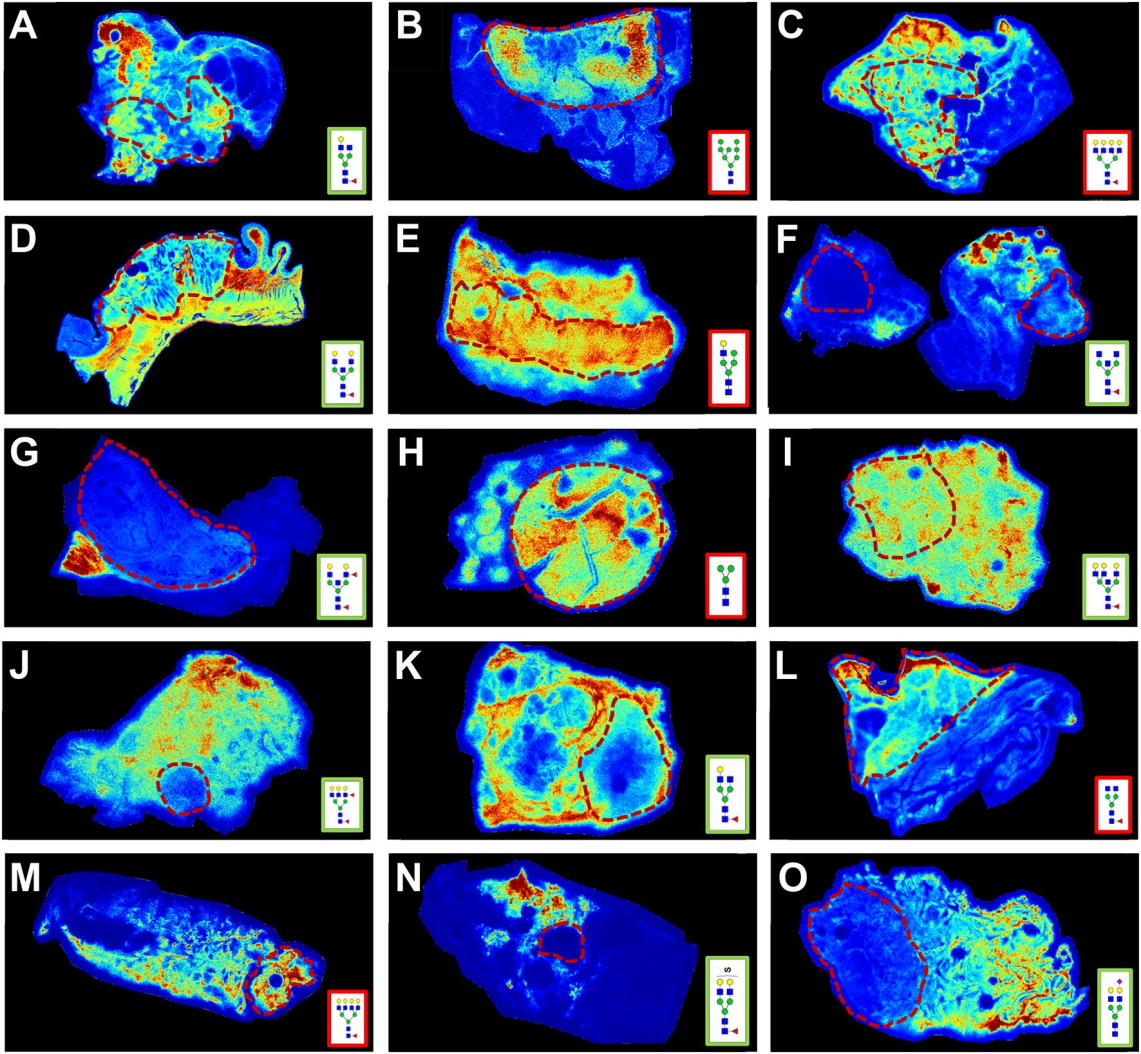
Relative abundance of *N-*glycans for each tissue type most altered in cancer. *N-*glycans with increased relative abundance in cancer (i.e., cancer-associated *N-*glycans) are outlined in red; *N-*glycans with decreased relative abundance in cancer (i.e., normal-associated *N-*glycans) are outlined in green. (**A**) Bladder (n = 4)—1647.5865 (Hex4dHex1HexNAc4). (**B**) Breast (n = 7)—1743.5810 (Hex8HexNAc2). (**C**) Cervix (n = 4)—2539.9037 (Hex7dHex1HexNAc6). (**D**) Colon (n = 10)—2012.7187 (Hex5dHex1HexNAc5). (**E**) Esophagus (n = 5)—1460.5020 (Hex5HexNAc3). (**F**) Gastric (n = 4)—1688.6130 (Hex3dHex1HexNAc5). (**G**) Kidney (n = 3)—2158.7766 (Hex5dHex2HexNAc5). (**H**) Liver (n = 4)—933.3170 (Hex3HexNAc2). (**I**) Lung (n = 10)—2377.8509 (Hex6dHex1HexNAc6). (**J**) Pancreas (n = 5)—2320.8294 (Hex6dHex2HexNAc5). (**K**) Prostate (n = 8)—1647.5865 (Hex4dHex1HexNAc4). (**L**) Sarcoma (n = 4)—1485.5337 (Hex3dHex1HexNAc4). (**M**) Skin (n = 5)—2539.9037 (Hex8HexNAc2). (**N**) Thyroid (n = 6)—1911.5859 (Hex5dHex1HexNAc4 + 2Na + SO_4_). (**O**) Uterus (n = 3)—1976.6666 (Hex7dHex1HexNAc6).

Because the TMA cores represent a small portion of a larger tissue source, representative full tissue slices for each of the 15 tissue types were processed for *N-*glycan MALDIMS. The H&E stains of each tissue with cancer regions highlighted are provided in Supplementary Fig. 21. In Fig. 3, histopathological distribution of the abundant *N-*glycans at 1809.64 *m/z* (Hex5dHex1HexNAc4) and 1663.58 *m/z* (Hex5HexNAc4) are shown with regard to tissue structure and cancer location (Fig. 3). These two glycans are generally seen in high relative abundance in the stromal regions surrounding the tumor. Using these tissue sections for further visualization, a representative *N-*glycan for every tissue type that was most altered in that cancer type was selected. To do this, *N-*glycans were ranked by their relative abundance in the tissue and their statistical significance, and the one with the highest significance that had the highest relative abundance was selected, shown in Fig. 4. For six of the tissues—breast, cervix, esophagus, liver, sarcoma, and skin—this was an *N-*glycan that was elevated in cancer, but the rest showed primarily decreases in glycan relative abundance (Fig. 4). Among these are several organs high in production of hormones (bladder, thyroid, pancreas, and prostate), and the kidney, which has large quantities of complex *N-*glycans in the glomeruli and tubules. In kidney tissue, the selected *N-*glycan that is high in normal tissue but low in cancer, a GlcNAc bisect, is distinctly located in the tubules of the kidney, where this type of *N-*glycan is known to be highly abundant^57^, but disappears almost entirely in the cancer that has invaded the tubule-rich region (Fig. 4G). Taken altogether, these results suggest that a major change between normal and cancerous tissue may be decreases in *N-*glycans related to normal function. Each tissue shown was used as a donor tissue for a normal and tumor core pair, and all the other *N-*glycans present in each tissue can be visualized in METASPACE. These differences for each tissue type are further highlighted in Supplementary Figs. 6–20.

### *N-*glycan fucosylation and sialylation

**Figure 5:**
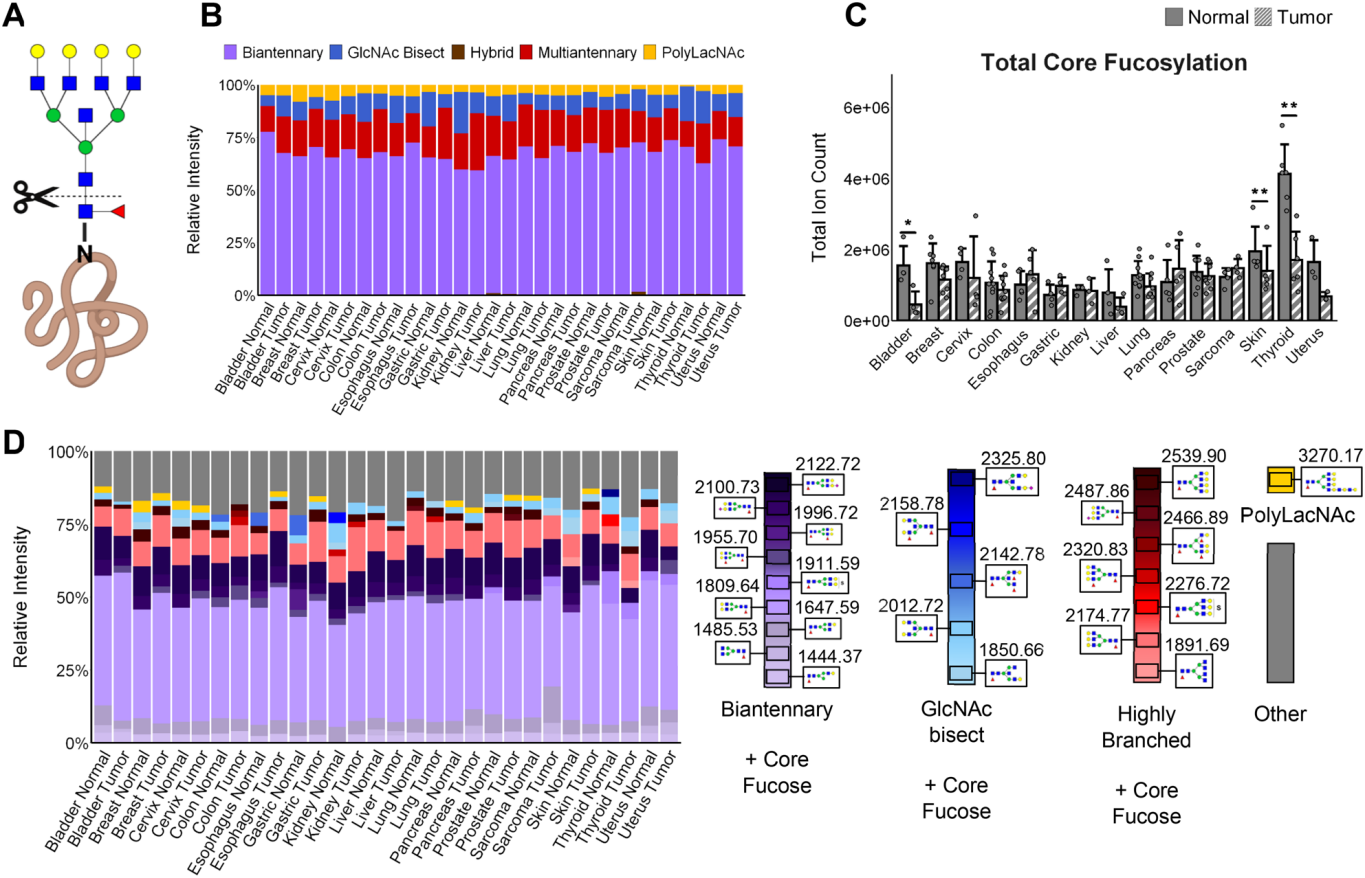
*N-*glycan core fucosylation (bladder n = 4, breast n = 7, cervix n = 4, colon n = 10, esophagus n = 5, gastric n = 4, kidney n = 3, liver n = 4, lung n = 10, sarcoma n = 4, skin n = 5, pancreas n = 5, prostate n = 8, thyroid n = 6, uterus n = 3). Significance is marked as follows: (*): p-value < 0.05; (**): p-value < 0.01; (***): p-value < 0.001; (****): p-value < 0.0001. Error bars represent one standard deviation. (**A**) Methodology used: EndoF3 severs core-fucosylated *N-*glycans between two core GlcNAc monomers. (**B**) Relative percent of core fucosylated *N-*glycans by type. (**C) **Total ion count of core-fucosylation by tissue type. Significance (student’s paired t-test) between normal and cancer is marked as follows: (*): p-value < 0.05; (**): p-value < 0.02; (***): p-value < 0.001. (**D**) Average relative intensities of top ten core-fucosylated *N-*glycans for each tissue type sorted by *N-*glycan type. Each column shows the top ten core-fucosylated *N-*glycans for that tissue, with the grey portion representing the sum of remaining *N-*glycans. The legend shows glycan structures and glycans are arranged by glycan type in a gradient from smallest *m/z* to largest.

In addition to using PNGase F to look at overall *N-*glycan types and intensities, we used other glycosidase digestions and chemical modification strategies to examine *N-*glycan fucosylated and sialylated structures in more depth. The glycosidase endoglycosidase F3 (EndoF3) was used instead of PNGase F to specifically identify the most abundant *N-*glycans with a core fucose structure^63,64^. EndoF3 recognizes core fucose N-glycans, and cleaves between the GlcNAc residues attached to asparagine, leaving a GlcNAc-Fuc product still attached to a protein (Fig. 5A). The released *N-*glycan has a mass shift loss of 349 m.u., which is then detected by MALDI MS. When applied to the TMAs, it was clear that the majority of core fucosylated *N-*glycans are biantennary and biantennary/bisecting structures (Fig. 5B). Example *N-*glycan image comparisons with PNGaseF vs. EndoF3 digestion are shown in Supplementary Fig. 3 for the most abundant bi-, tri- and tetra-antennary *N-*glycans, highlighting the shift of -349 m.u. for detected core fucosylated species. The proportion of core fucosylated tri- and tetra-antennary species decreases in larger mass *N-*glycans, demonstrating mixtures of core and outer arm fucosylation across tissues. Cumulatively, total core fucosylation was not increased in cancers across the board, which may indicate that it is specific *N-*glycans being core fucosylated that is the important factor in cancer, rather than simply overall core fucosylation (Fig. 5C). In fact, in all cancer types with a significant difference between normal and cancer, there was a significant decrease in total core fucosylation compared to the healthy tissue (Fig. 5C). Looking at relative percentages of glycan types that were core fucosylated, there does appear to be an increase in multiantennary *N-*glycans with core fucosylation in many cancers, which is not surprising given that both multiantennary *N-*glycans and core fucosylation have been linked to cancer (Fig. 5B, D)^26,30,31^. Using the graphic representation shown in Fig. 2, the top 10 most abundant core fucosylated *N-*glycans detected in each tissue types are presented in Fig. 5D.

**Figure 6:**
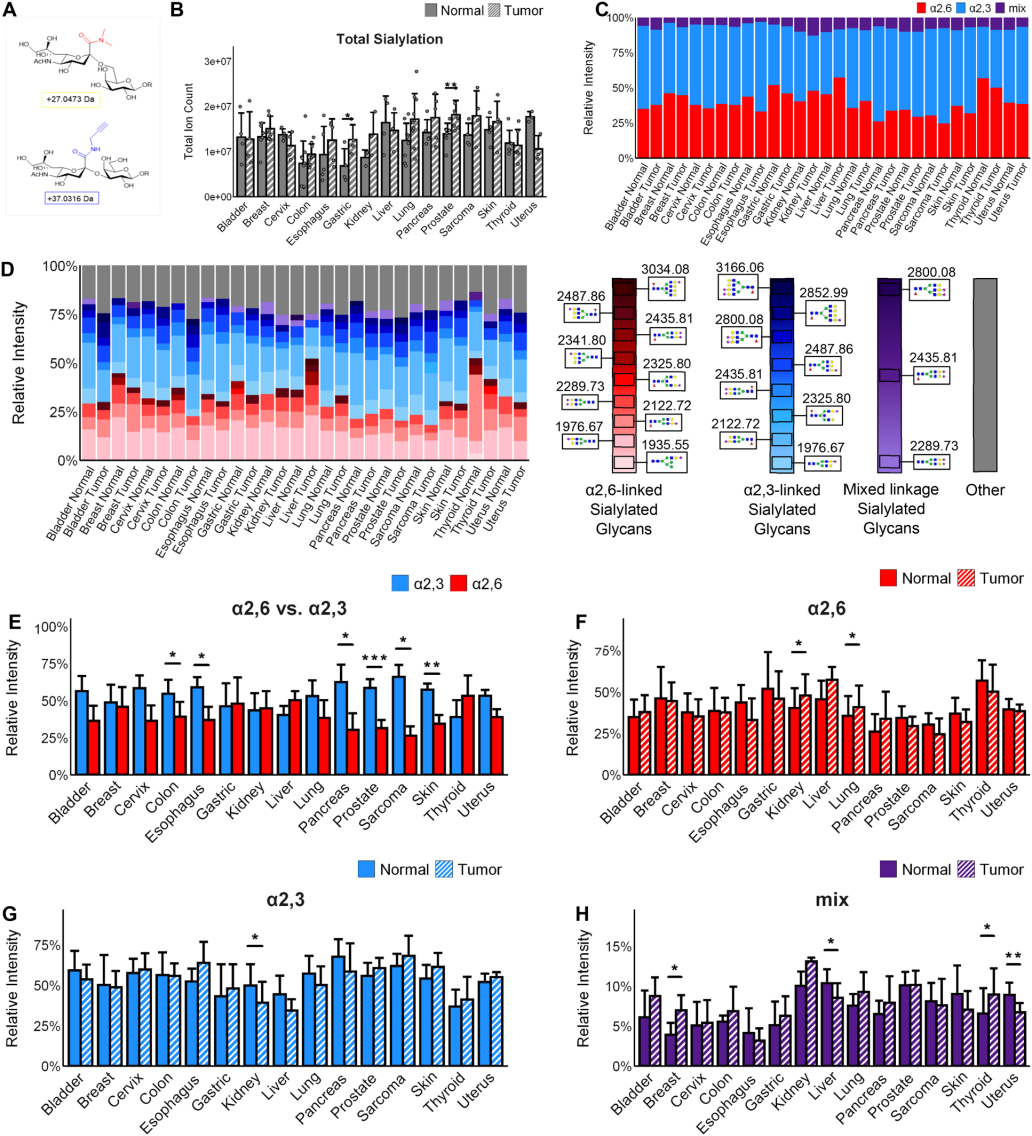
Relative abundance and distribution of sialylated *N-*glycans (bladder n = 4, breast n = 7, cervix n = 4, colon n = 10, esophagus n = 5, gastric n = 4, kidney n = 3, liver n = 4, lung n = 10, sarcoma n = 4, skin n = 5, pancreas n = 5, prostate n = 8, thyroid n = 6, uterus n = 3). Significance is marked as follows: (*): p-value < 0.05; (**): p-value < 0.01; (***): p-value < 0.001; (****): p-value < 0.0001. Error bars on bar charts represent one standard deviation. Error bars on boxplots represent the quartiles. (**A**) Linkage-specific amino groups are added to sialic acid with “CLICK chemistry” to stabilize sialylation and distinguish between α2,6 and α2,3 linkages. (**B**) Total ion count of sialylated *N-*glycan relative abundance by tissue type. (**C**) Comparison of α2,6 and α2,3 linkage totals by tissue type. (**D**) Average relative intensities of the top ten sialylated *N-*glycans in each tissue by linkage type. Each column shows the top ten sialylated *N-*glycans for that tissue, with the grey portion representing the sum of remaining *N-*glycans. Legend shows glycan structures and glycans are arranged by sialic acid linkage type in a gradient from smallest *m/z* to largest. (**E**) Overall relative proportion of sialic acid linkages seen in each tissue type. (**F**) Comparison of α2,6 linkage relative abundances between normal and cancerous tissues. (**G**) Comparison of α2,3 linkage relative abundances between normal and cancerous tissues. (**H**) Comparison of mixed α2,6/α2,3 sialylation between normal and cancerous tissues of each type.

Sialic acid is also disease and cancer-relevant^33–36^, and an amidation stabilization chemistry termed AAXL (Alkyne-Amidation Xtra Linker) was used to differentiate between α2,3 and α2,6 sialic acid linkages (Fig. 6A)^43^. This approach introduces a + 27 m.u. dimethylamine group for α2,6 linkages, and a + 37 m.u. alkyne amine group for α2,3. The sialic acid linkage distributions were assessed by *N-*glycan IMS after amidation and PNGaseF release, and the total amount of sialylated *N-*glycan intensities were summed for each tissue type (Fig. 6B). The patterns of total sialylated *N-*glycan relative abundance were distinct from overall *N-*glycan intensity, as the sarcoma tumor and pancreas and lung normal tissues showed the highest overall levels of sialylation (Fig. 6B). Gastric and prostate cancer showed significantly more sialylation than their normal tissue counterparts (Fig. 6B).

Overall, there were more α2,3 linked sialic acids seen in these tissues, both in the top abundant *N-*glycans and in total sialylated *N-*glycan proportion (Fig. 6C–F). In Fig. 6D, the graphical distributions of the top ten most abundant sialylated *N-*glycans for each tissue type are shown. When two similar *N-*glycans were highly abundant, the one possessing a core fucose was most often seen to have an α2,3 sialic acid linkage, while the one without typically had a more abundant α2,6 linkage (Fig. 6D). Notably, the highest detected sialylated *N-*glycan with the α2,3 linkage was Hex5dHex1HexNAc4NeuAc1 at *m/z* 2122.7, while the highest detected *N-*glycan with the α2,6 sialic acid linkage was the non-fucosylated Hex5HexNAc4NeuAc1 at *m/z* 1976.7 (Fig. 6D). These are the sialylated versions of the most abundant *N-*glycans seen previously at 1809 and 1663 *m/z*, respectively. Of the *N-*glycans with two or more sialic acids and mixed linkages, only three were seen in the top ten for any tissues, and two of these were the di-sialylated versions of the overall two most abundant sialylated *N-*glycans (Fig. 6D). Roughly half the tumors showed a tendency for more of one linkage type compared to the corresponding healthy tissue, and of these, there was a significantly higher α2,6 relative abundance in kidney and liver cancer compared to normal tissue and lower α2,3 relative abundance in kidney cancer compared to normal tissue (Fig. 6F, G). Additionally, a decrease was often seen in one linkage when the other was increased between normal and cancerous tissue—significantly so in the case of kidney tissue (Fig. 6F, G). Most of the tumor types showed some change in mixed linkage multi-sialylated *N-*glycans, with significant differences between normal and cancerous in breast, liver, thyroid, and uterus tissues, though these changes did not correlate to the single linkage changes (Fig. 6H). This indicates that linkage-specific sialylation varies across cancer types and plays roles in the different pathways of cancer development. This once again demonstrates that there are few *N-*glycan changes that are universal across cancers, but all such changes play important roles in their relative cancers.

## Discussion

The cumulative *N-*glycan tissue data illustrates notable trends in overall *N-*glycan relative abundances and changes between fifteen normal tissues and their corresponding tumors. The data generated illustrates how every tissue type has key *N-*glycans, and cancer-associated *N-*glycan changes differ between tissue types. Mapping of tissue *N-*glycans and deciphering tissue-specific protein *N*-glycosylation has the potential to uncover increasingly informative alterations in disease and the biological behaviors behind it. Finding the distinct and highly abundant *N-*glycans for different tissue types that are drastically altered in cancer may uncover disease mechanisms or even prove to be early indicators of tumor aggressiveness. Such research for individual cancers is already a major part of this field, but only a handful of tissues have been studied in depth. Thus, our initial atlasing efforts should encourage further research for identifying *N-*glycan biomarkers. While most research focuses on how glycans are altered in disease states, understanding healthy tissue behavior will be just as informative, and studies such as this one will hopefully lead to more such research of non-diseased glycan roles. For future *N-*glycan tissue studies, understanding the underlying mechanisms of changes among individuals, racial and/or gender-specific changes, and changes over time is already underway for breast tissues^65^. These aspects are already well-studied for changes in *N-*glycan compositions of circulating immunoglobulin glycoproteins in blood^7,8,66^.

There are some limitations of the present data that should be noted. One limitation is the overall sample size, which is too small to use the data for clinical conclusions or biomarker considerations. Rather, our atlas is intended be used as a reference resource for future larger tissue glycomic studies that address these questions, as well as other research uses. Another limitation is that the data was generated by only one mass spectrometry method and ionization source. There are many approaches to analyze and identify *N-*glycans by mass spectrometry approaches using different ionization sources, tandem liquid chromatography, and capillary electrophoresis workflows^36,37,39^. It is possible to determine differential relative abundance levels of tissue *N-*glycans using other methods, and this MS imaging approach is most effective at identifying the most abundant *N*-glycans. However, the *N-*glycan imaging mass spectrometry approach will always have the advantage of characterizing spatial distributions and links to histopathology features. We attempted to report on most of the major organs in the human body, but an unfortunate omission is brain tissue. Many brain tumor resections involve minimal removal of healthy/non-tumor regions, so creating a multi-sample cohort was not feasible when the TMAs were created. There are several recent reviews on brain glycosylation^67–71^, generally for neurodegenerative conditions like Alzheimer’s disease, with notable descriptions of abundant biantennary bisecting *N-*glycans unique to healthy brain. Comparative *N-*glycan imaging mass spectrometry studies have also been reported for healthy versus neurodegenerative disease brain tissues^72–74^. Using unpublished normal human brain data from our group and the processing workflows described herein, the most abundant *N-*glycans detected are consistent with the published studies in that bisecting biantennary glycans, their precursors, and oligomannose species were identified.

Lastly, a long-standing problem for any type of analysis of *N-*glycan structures is the glycan diversity created from many possible isomers, ones that are present in even the simplest oligomannose structures. There are at a minimum over 2000 *N-*glycan structures, and these arise from different combinations of nine primary monosaccharide species, anomeric and linkage differences, branching/extensions, and other modifications like sulfate or phosphate additions^12,14^. The 97 *N-*glycans included in this report are therefore basic compositions, with each specie likely having many possible isomer structures. There are certainly additional higher mass *N-*glycans present in these tissues that exceeded the detection limit of the mass spectrometer. Using endoF3 to identify core versus outer arm fucose isomers and AAXL stabilization to label α2,3 and α2,6 isomers reduces the amount of isomeric complexity in our dataset; however, more advanced separation tools like ion mobility mass spectrometry instruments are required to further deconvolute the remaining isomeric species.

As research efforts continue to define the human *N-*glycome, sharing of data and data accessibility become increasingly important. Tools to share data throughout the field have begun to be developed and integration of multi-omic and multi-modal data sets will need to be integrated. Glycan data resources developed by the GlyCosmos and GlyGen projects provide web accessible portals for glycoscience containing standards, repositories, and a variety of data resources on glycans and the molecules and pathways they play a role in modifying^46^. There are also several glycan structure resources that have been developed and continue to increase in information and usage, including GlyTouCan^75^, which is the international glycan repository, and NGlycDB, an *N-*glycan specific database used in METASPACE^44,45^. All our data from this research will be available on the METASPACE platform, with *N-*glycan annotations currently created through the NGlycDB database, and soon to be linked with resources in GlyCosmos ^46,75^. New *N-*glycan IMS data from other human tissues, like the aforementioned brain studies, will be uploaded to these sites as studies mature.

Additionally, the TMA used here has broad usefulness for glycomic profiling in a broad range of profiling methods in future research, including additional enzymes as well as new multiplexed techniques like MALDI- immunohistochemistry^76^. Current ongoing work in the lab using these TMA slides includes an in-depth investigation of poly-LacNAc-ylation and sulfation, as there are several highly abundant putative sulfated glycans detected in these TMAs. As more techniques continue to be developed for MALDI tissue profiling, TMAs such as this one will be vital for better vetting of techniques and efficient analysis for deeper knowledge in research studies.

In summary, we report an overview of fifteen major human tissues and a broad compositional assessment of their *N-*glycan profiles, comparative relative abundances and cancer associated changes. The data provided for a tissue-specific human *N-*glycome is only a starting point and reference for future work, with the need for other methodologies to be utilized. The spatial glycomic data available in METASPACE in conjunction with this study may lead to more discoveries with this data. *N-*glycomics is a valuable tool for increasing our understanding of cellular biology and promises important discoveries and clinical applications in the near future.

## Materials and methods

### Materials

MALDI matrix α-cyano-4-hydroxycinnamic acid (CHCA) was obtained from Sigma-Aldrich (St. Louis, MO). Tissue-tack glass histology slides, acetonitrile (HPLC grade), citraconic anhydride, H2O (HPLC grade), and xylenes (histological grade) were obtained from ThermoFisher Scientific (Waltham, MA). Recombinant peptide *N-*glycosidase F (PNGase F PRIME), Endo-glycosidase F3 (EndoF3 PRIME), and sialidase were obtained from *N-*zyme Scientific (Doylestown, PA).

### Methods

#### Formalin fixed paraffin embedded tissues and tissue microarrays

Two custom made tissue microarray blocks were created following selection of 89 tissue blocks out of 125 candidates, representing 20 human tumor types. Each tissue selected was annotated by a pathologist for core targets that represented areas of tumor and areas on adjacent normal regions. The tissue of origin and description of tumor type for each core (n = 198) in the TMAs are provided in Supplementary Table 1. The tissue microarrays were created by the Medical University of South Carolina’s Hollings Cancer Center Biorepository and Tissue Analysis Shared Resource in accordance with the National Cancer Institute’s Best Practices for Biospecimen Resources. All tissues were archival, all donors were anonymous to the investigators, and any personal identifiers were not included. Use of the tissues was approved by the Institutional Review Board at the Medical University of South Carolina. Each core is approximately 1 mm in diameter and the cores are organized as tumor and normal pairs. For data analysis presented in this report, only tissue types represented by at least three normal and tumor cores were used. Fifteen normal/tissue types met this criterion: bladder n = 4, breast n = 7, colon n = 10, kidney n = 3, liver n = 4, lung n = 10, pancreas n = 5, prostate n = 8, thyroid n = 6, uterus n = 3, cervix n = 4, esophagus n = 5, gastric n = 4, skin (non-melanoma) n = 5, and sarcoma n = 4. Data obtained for glioma, head and neck, ovary, testes and melanoma tissues, which had two or less core pairs, were not included in the comparative analyses. Additionally, representative full tissue slice donor blocks were selected for each of the 15 tumor types and processed for *N-*glycan imaging mass spectrometry analysis.

#### *N-*glycan MALDI imaging mass spectrometry of FFPE tissue slides

The tissue TMA slides and full slice tissue slides were prepared for *N-*glycan imaging mass spectrometry analysis using the same workflow. A standardized tissue preparation workflow was followed, which has been previously published^40^, and described in the recent analysis of prostate^77,78^ and pancreatic^34^ cancer FFPE tissues. Briefly, tissue slides were dewaxed and rehydrated, followed by antigen retrieval in citraconic anhydride buffer, pH 3 for 30 min in a decloaking chamber at 95 °C. After buffer exchange and drying in a desiccator, 15 passes of PNGaseF PRIME enzyme at 0.1 µg/µL was applied as a molecular coating to the tissue slides at a rate 25 µL/min with a velocity of 1200 mm/min and a 3 mm offset at 10 psi and 45 °C using an M5 Sprayer (HTX Technologies, Chapel Hill, NC). Slides were incubated in prewarmed humidity chambers for 2 h at 37 °C for deglycosylation. After PNGaseF digestion, 7 mg/mL CHCA matrix in 50% ACN/0.1% TFA was applied to the deglycosylated slides at a rate of 100 µL/min with a velocity of 1300 mm/min and a 2.5 mm offset at 10 psi and 79 °C using the same sprayer. After matrix application slides were desiccated until analysis.

Two MALDI instruments were used in the study, a Solarix dual source 7 T MALDI-FTICR mass spectrometer and a timsTOF Flex MALDI-QTOF mass spectrometer (Bruker Corporation, Billerica, MA), operated as previously described^34^. The Solarix MALDI-FTICR was used for the TMA imaging and the MALDI-QTOF was used to image the larger tissue samples. Data was collected at mass ranges of 700–4000 *m/z* in positive ion mode, at laser spot sizes of 20–25 µm, 300 laser shots per pixel and 40 µm raster. Post-acquisition, spectra were imported to SCiLS Lab software (Bruker Corporation, Billerica, MA) for processing imaging mass spectrometry experiments. *N-*glycan spectra were normalized to total ion count. Spectra were annotated by matching glycan peak m/z values to an established in-house *N-*glycan database that relies on the reproducible and highly accurate mass determinations obtained from MALDI-QTOF and MALDI-FTICR instrumentation ^34,77,78^. Structural assignments were based off of cumulative prior characterizations by MALDI-TOF–MS/MS collision induced dissociation^34^, reversed-phase liquid chromatography-coupled tandem mass spectrometry^79^, use of endo F3^42^ and sialic acid stabilization by amidation^43,79^. Structural codes linked with the GlyTouCan database^75^ were also added.

#### Core fucosylation and sialylation analysis

For core fucosylation detection, the enzyme Endo F3 was used instead, using the above protocol but with pH 4 water to suspend the enzyme ^42,64^. To detect sialylation, Alkyne-Amidation Xtra Linker (AAXL) amidation-amidation chemistry was performed to stabilize α2,3 and α2,6 linked sialic acids and add either dimethylamine (+ 27 m.u. for α2,6) or propargylamine (+ 37 m.u. for α2,3) to the sialic acids to create a detectable isomer-specific mass shift^43,80^.

The FFPE tissue slides were heated at 60 °C for 1 h, then dewaxed and rehydrated with xylenes and a series of ethanol and water washes and dried in a desiccator for 30 min. Each slide was treated with 200 μL of the first AAXL reaction solution and incubated at 60 °C for 1 h. Slides were washed with DMSO and vacuum aspiration, then treated with 200 μL of the second AAXL reaction solution and incubated at 60 °C for 2 h. Slides were then washed with ethanol, Carnoy’s solution (60% ethanol, 30% chloroform and 10% glacial acetic acid), TFA and water^43^.

Following amidation, PNGaseF digestion and subsequent *N-*glycan MALDI IMS was done using the standard protocol described above. After imaging mass spectrometry, matrix was removed for H&E staining and high-resolution image scanning (Hamamatsu NanoZoomer 2.0RS).

### Data processing and statistical analysis

Mass spectra were imported into SCiLS Lab 2022b Pro (Bruker), normalized to total ion count, and peak selected for *N-*glycans based on theoretical *m/z* values. Area under the peak intensity data for each glycan was determined and exported for further analysis. The intensity values for each analysis and the *N-*glycan structural compositions are provided in Supplementary Tables 2 and 3. Data organization, statistical analysis, clustering analysis, and graphing were performed using R Statistical Software(v4.2.3)^81,82^. Statistical analysis was performed using R\rstatix package^83^. Figures were generated in R\ggpubr^84^. Clustering analysis was performed using R\stats and R\ggdendro^85^, using the Euclidean distances to form a linkage matrix.

To normalize the data further and account for differences in protein concentrations between tissue types, *N-*glycan relative intensities were calculated by taking the total *N-*glycan intensity for the region in question and dividing all the individual *N-*glycan intensities by this number for each. Paired two-tailed t-tests were performed on each tissue type to compare normal tissue to tumor tissue for each glycan, using a p-value for significance of < 0.05, with additional cutoffs for higher significance at p = 0.01, p = 0.001, and p = 0.0001. *N-*glycans were analyzed both on an individual basis as well as by structural groups formed using our glycan structure database and taking the sum of glycans in each category for each sample. The top 20 highest relative abundance *N-*glycans for each tissue type were selected for further analysis, based on peak intensity values, and the *N-*glycans were grouped based on general structure categories, with specific attention given to separating those with a core fucose. For sialylated and core-fucosylated *N-*glycans, the top 10 *N-*glycans of each were selected for further analysis and grouped by linkages.

### Supplementary Information

Supplementary Figures.

Supplementary Legends.

Supplementary Table 1.

Supplementary Table 2.

Supplementary Table 3.

## Data Availability

The data used in this study are available in the source data for this article and in the METASPACE data repository (https://metaspace2020.eu/api_auth/review?prj=e3df0604-8d24-11ed-b629-0b81a3feb0bc&token=aZFyC9cL3SO1).

## References

[1] National Human Genome Research Institute. *The**Human**Genome**Project*. https://www.genome.gov/human-genome-project (2022).

[2] Hood L, Rowen L (2013). The Human Genome Project: Big science transforms biology and medicine. Genome Med..

[3] Human Proteome Project. *Human**Proteome**Project:**HPP**Progress**to**Date*. https://www.hupo.org/human-proteome-project/hpp-progress-to-date (2022).

[4] Uhlén M (2015). Tissue-based map of the human proteome. Science.

[5] Rozenblatt-Rosen O, Stubbington MJT, Regev A, Teichmann SA (2017). The Human Cell Atlas: From vision to reality. Nature.

[6] Wishart DS (2022). HMDB 5.0: The Human Metabolome Database for 2022. Nucleic Acids Res..

[7] TrbojevicAkmacic I (2022). High-throughput glycomic methods. Chem. Rev..

[8] Shkunnikova S (2023). IgG glycans in health and disease: Prediction, intervention, prognosis, and therapy. Biotechnol. Adv..

[9] Blaschke CRK (2021). Glycan imaging mass spectrometry: Progress in developing clinical diagnostic assays for tissues, biofluids, and cells. Clin. Lab. Med..

[10] Ruhaak LR, Miyamoto S, Lebrilla CB (2013). Developments in the identification of glycan biomarkers for the detection of cancer. Mol. Cell. Proteom..

[11] McDowell CT, Lu X, Mehta AS, Angel PM, Drake RR (2023). Applications and continued evolution of glycan imaging mass spectrometry. Mass Spectrom. Rev..

[12] Cummings RD (2009). The repertoire of glycan determinants in the human glycome. Mol. BioSyst..

[13] Moremen KW, Tiemeyer M, Nairn AV (2012). Vertebrate protein glycosylation: Diversity, synthesis and function. Nat. Rev. Mol. Cell Biol..

[14] Stanley, P., Moremen, K. W., Lewis, N. E., Taniguchi, N. & Aebi, M. *Essentials**of**Glycobiology* (eds. Varki, A. *et**al.*). 103–116 (Cold Spring Harbor Laboratory Press, 2022) *Copyright**©**2022**The**Consortium**of**Glycobiology**Editors,**La**Jolla,**California*; *Published**by**Cold**Spring**Harbor**Laboratory**Press*. 10.1101/glycobiology.4e.9 (all rights reserved).

[15] Varki A, Gagneux P (2015). Biological Functions of Glycans.

[16] van Kooyk Y, Rabinovich GA (2008). Protein-glycan interactions in the control of innate and adaptive immune responses. Nat. Immunol..

[17] Dube DH, Bertozzi CR (2005). Glycans in cancer and inflammation—Potential for therapeutics and diagnostics. Nat. Rev. Drug Discov..

[18] Varki A (2016). Biological roles of glycans. Glycobiology.

[19] Pinho SS, Reis CA (2015). Glycosylation in cancer: Mechanisms and clinical implications. Nat. Rev. Cancer.

[20] Smith BA, Bertozzi CR (2021). The clinical impact of glycobiology: Targeting selectins, Siglecs and mammalian glycans. Nat. Rev. Drug Discov..

[21] Munkley J, Elliott DJ (2016). Hallmarks of glycosylation in cancer. Oncotarget.

[22] Rini, J. M., Moremen, K. W., Davis, B. G. & Esko, J. D. Glycosyltransferases and glycan-processing enzymes. In *Essentials**of**Glycobiology* [Internet]. 4th ed. (2022).

[23] Boyaval F (2022). High-mannose *N*-glycans as malignant progression markers in early-stage colorectal cancer. Cancers.

[24] de Leoz MLA (2011). High-mannose glycans are elevated during breast cancer progression. Mol. Cell. Proteom..

[25] Park DD (2020). Metastasis of cholangiocarcinoma is promoted by extended high-mannose glycans. Proc. Natl. Acad. Sci..

[26] Ščupáková K (2021). Clinical importance of high-mannose, fucosylated, and complex N-glycans in breast cancer metastasis. JCI Insight.

[27] Chen Q, Tan Z, Guan F, Ren Y (2020). The essential functions and detection of bisecting GlcNAc in cell biology. Front. Chem..

[28] Kohler RS (2016). Epigenetic activation of MGAT3 and corresponding bisecting GlcNAc shortens the survival of cancer patients. Oncotarget.

[29] Kawade H (2021). Tissue-specific regulation of HNK-1 biosynthesis by bisecting GlcNAc. Molecules.

[30] Herrera H (2019). Core-fucosylated tetra-antennary N-glycan containing a single *N*-acetyllactosamine branch is associated with poor survival outcome in breast cancer. Int. J. Mol. Sci..

[31] West CA (2018). N-linked glycan branching and fucosylation are increased directly in Hcc tissue as determined through in situ glycan imaging. J. Proteome Res..

[32] Scott DA (2019). Increases in tumor *N*-glycan polylactosamines associated with advanced HER2-positive and triple-negative breast cancer tissues. Proteom.-Clin. Appl..

[33] Marciel, M. P., Haldar, B., Hwang, J., Bhalerao, N. & Bellis, S. L. *Advances**in**Cancer**Research.* Vol. 157. 123–155 (Elsevier, 2023). 10.1016/bs.acr.2022.07.003 PMC11342334 36725107

[34] McDowell CT (2021). Imaging mass spectrometry and lectin analysis of N-linked glycans in carbohydrate antigen-defined pancreatic cancer tissues. Mol. Cell. Proteom..

[35] Munkley J (2022). Aberrant sialylation in cancer: Therapeutic opportunities. Cancers.

[36] Xu C (2021). Recent advances in understanding the roles of sialyltransferases in tumor angiogenesis and metastasis. Glycoconjug. J..

[37] De Haan N, Wuhrer M, Ruhaak L (2020). Mass spectrometry in clinical glycomics: The path from biomarker identification to clinical implementation. Clin. Mass Spectrom..

[38] Ruhaak LR, Xu G, Li Q, Goonatilleke E, Lebrilla CB (2018). Mass spectrometry approaches to glycomic and glycoproteomic analyses. Chem. Rev..

[39] Riley NM, Bertozzi CR, Pitteri SJ (2021). A pragmatic guide to enrichment strategies for mass spectrometry-based glycoproteomics. Mol. Cell. Proteom..

[40] Drake RR, Powers TW, Norris-Caneda K, Mehta AS, Angel PM (2018). In situ imaging of *N*-glycans by MALDI imaging mass spectrometry of fresh or formalin-fixed paraffin-embedded tissue. Curr. Protoc. Protein Sci..

[41] Clift CL, Drake RR, Mehta A, Angel PM (2021). Multiplexed imaging mass spectrometry of the extracellular matrix using serial enzyme digests from formalin-fixed paraffin-embedded tissue sections. Anal. Bioanal. Chem..

[42] West CA, Liang H, Drake RR, Mehta AS (2020). New enzymatic approach to distinguish fucosylation isomers of N-linked glycans in tissues using MALDI imaging mass spectrometry. J. Proteome Res..

[43] Lu X (2023). Bioorthogonal chemical labeling probes targeting sialic acid isomers for *N*-glycan MALDI imaging mass spectrometry of tissues, cells, and biofluids. Anal. Chem..

[44] Palmer A (2017). FDR-controlled metabolite annotation for high-resolution imaging mass spectrometry. Nat. Methods.

[45] Veličković D (2021). Rapid automated annotation and analysis of *N*-glycan mass spectrometry imaging data sets using NGlycDB in METASPACE. Anal. Chem..

[46] Yamada I (2020). The GlyCosmos portal: A unified and comprehensive web resource for the glycosciences. Nat. Methods.

[47] Baudry N (1996). Dityrosine bridge formation and thyroid hormone synthesis are tightly linked and are both dependent on *N*-glycans. FEBS Lett..

[48] Mallet B (1995). *N*-glycans modulate in vivo and in vitro thyroid hormone synthesis: Study at the *N*-terminal domain of thyroglobulin. J. Biol. Chem..

[49] Ząbczyńska M, Kozłowska K, Pocheć E (2018). Glycosylation in the thyroid gland: Vital aspects of glycoprotein function in thyrocyte physiology and thyroid disorders. Int. J. Mol. Sci..

[50] Stanley, P., Wuhrer, M., Lauc, G., Stowell, S. R. & Cummings, R. D. Structures common to different glycans. In *Essentials**of**Glycobiology* [Internet]. 4th ed. (2022).

[51] Cheng P-W, Davidson S, Bhat G (2020). Markers of malignant prostate cancer cells: Golgi localization of α-mannosidase 1A at GM130-GRASP65 site and appearance of high mannose *N*-glycans on cell surface. Biochem. Biophys. Res. Commun..

[52] Everest-Dass AV (2016). N-glycan MALDI imaging mass spectrometry on formalin-fixed paraffin-embedded tissue enables the delineation of ovarian cancer tissues. Mol. Cell. Proteom..

[53] Heijs B (2020). Molecular signatures of tumor progression in myxoid liposarcoma identified by *N*-glycan mass spectrometry imaging. Lab. Invest..

[54] Liang Y (2019). Stage-associated differences in the serum *N*-and *O*-glycan profiles of patients with non-small cell lung cancer. Clin. Proteom..

[55] DelaCourt A (2021). N-glycosylation patterns correlate with hepatocellular carcinoma genetic subtypes. Mol Cancer Res.

[56] Ryczko MC (2016). Metabolic reprogramming by hexosamine biosynthetic and Golgi *N*-glycan branching pathways. Sci. Rep..

[57] Drake RR (2020). Defining the human kidney *N*-glycome in normal and cancer tissues using MALDI imaging mass spectrometry. J. Mass Spectrom..

[58] Kinoshita M (2014). Common glycoproteins expressing polylactosamine-type glycans on matched patient primary and metastatic melanoma cells show different glycan profiles. J. Proteome Res..

[59] Mitsui Y (2012). Comparative studies on glycoproteins expressing polylactosamine-type *N*-glycans in cancer cells. J. Pharmaceut. Biomed. Anal..

[60] Wuhrer M (2019). Paucity of paucimannosylation revoked. Proteomics.

[61] Chatterjee S (2019). Protein paucimannosylation is an enriched *N*-glycosylation signature of human cancers. Proteomics.

[62] Chen, M. *et**al.**Comparative**Site-Specific**N-Glycoproteome**Analysis**Reveals**Aberrant**N-Glycosylation**and**Gives**New**Insights**into**Mannose-6-Phosphate**Pathway**in**Cancer* (2022). 10.1038/s42003-023-04439-4 PMC9839730 36639722

[63] DelaCourt AT, Liang H, Drake RR, Angel PM, Mehta AS (2022). Novel combined enzymatic approach to analyze nonsialylated N-linked glycans through MALDI imaging mass spectrometry. J. Proteome Res..

[64] West, C. A. *et**al.**Mass**Spectrometry**of**Glycoproteins:**Methods**and**Protocols* (ed. Delobel, A.). 303–316 (Springer, 2021).

[65] Rujchanarong D (2022). Metabolic links to socioeconomic stresses uniquely affecting ancestry in normal breast tissue at risk for breast cancer. Front. Oncol..

[66] Pučić M (2011). High throughput isolation and glycosylation analysis of IgG—Variability and heritability of the IgG glycome in three isolated human populations. Mol. Cell. Proteom..

[67] Helm J (2021). Bisecting Lewis X in hybrid-type *N*-glycans of human brain revealed by deep structural glycomics. Anal. Chem..

[68] Klarić TS, Lauc G (2022). The dynamic brain *N*-glycome. Glycoconjug. J..

[69] Lee J (2020). Spatial and temporal diversity of glycome expression in mammalian brain. Proc. Natl. Acad. Sci..

[70] Williams SE (2022). Mammalian brain glycoproteins exhibit diminished glycan complexity compared to other tissues. Nat. Commun..

[71] Tang X (2023). Transcriptomic and glycomic analyses highlight pathway-specific glycosylation alterations unique to Alzheimer’s disease. Sci. Rep..

[72] Conroy LR, Hawkinson TR, Young LE, Gentry MS, Sun RC (2021). Emerging roles of N-linked glycosylation in brain physiology and disorders. Trends Endocrinol. Metab..

[73] Hawkinson TR (2022). In situ spatial glycomic imaging of mouse and human Alzheimer's disease brains. Alzheimer's Dement..

[74] Hasan MM (2021). Mass spectrometry imaging for glycome in the brain. Front. Neuroanat..

[75] Fujita A (2020). The international glycan repository GlyTouCan version 3.0. Nucleic Acids Res..

[76] Dunne J (2023). Evaluation of antibody-based single cell type imaging techniques coupled to multiplexed imaging of *N*-glycans and collagen peptides by matrix-assisted laser desorption/ionization mass spectrometry imaging. Anal. Bioanal. Chem..

[77] Butler W (2023). Rewiring of the *N*-glycome with prostate cancer progression and therapy resistance. npj Precis. Oncol..

[78] Blaschke CRK (2021). Direct *N*-glycosylation profiling of urine and prostatic fluid glycoproteins and extracellular vesicles. Front. Chem..

[79] Holst S, Belo AI, Giovannetti E, van Die I, Wuhrer M (2017). Profiling of different pancreatic cancer cells used as models for metastatic behaviour shows large variation in their N-glycosylation. Sci. Rep..

[80] Holst S (2016). Linkage-specific in situ sialic acid derivatization for *N*-glycan mass spectrometry imaging of formalin-fixed paraffin-embedded tissues. Anal. Chem..

[81] R Core Team, R. *R:**A**Language**and**Environment**for**Statistical**Computing*. (R Core Team, 2013).

[82] Wickham H (2019). Welcome to the Tidyverse. J. Open Source Softw..

[83] Alboukadel, K. *rstatix:**Pipe-Friendly**Framework**for**Basic**Statistical**Tests* (2023).

[84] Alboukadel, K. *ggpubr:**'ggplot2'**Based**Publication**Ready**Plots* (2023).

[85] De Vries, A. & Ripley, B. D. *ggdendro:**Create**Dendrograms**and**Tree**Diagrams**Using’ggplot2’.**R**Package**Version**0.1–20* (2016).

