# Supplementary material for: An N-glycome tissue atlas of 15 human normal and cancer tissue types determined by MALDI-imaging mass spectrometry
Source: Sci Rep. 2024 Jan 4;14:489. doi: 10.1038/s41598-023-50957-w (PMC10766640; doi:10.1038/s41598-023-50957-w)
Supplement: Supplementary file 1 — Supplementary Figures. [file 41598_2023_50957_MOESM1_ESM.pdf]

Rank	Glycan m/z	Structure	Rank	Glycan m/z	Structure
1	1809.6393	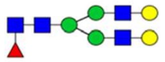	11	1850.6659	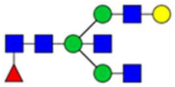
2	1663.5814	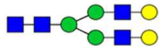	12	1419.4754	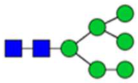
3	2174.7715	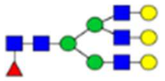	13	1444.5071	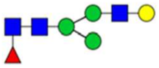
4	2012.7187	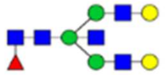	14	1485.5337	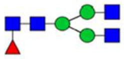
5	2122.7245	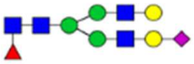	15	1905.6338	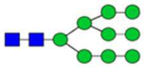
6	1976.6666	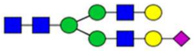	16	1257.4226	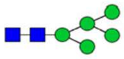
7	1911.5859	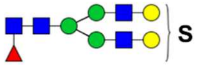	17	2276.7180	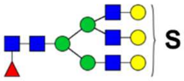
8	1647.5865	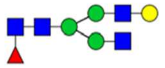	18	1581.5282	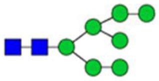
9	2539.9037	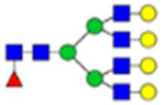	19	1298.4492	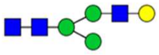
10	1743.5810	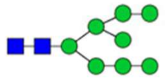	20	2377.8509	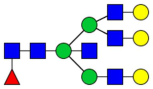

Supplementary Fig. 1. Top 20 global glycans. *N*-glycans comprising the top 20 glycans when all tissue data was summed, ranked by abundance.

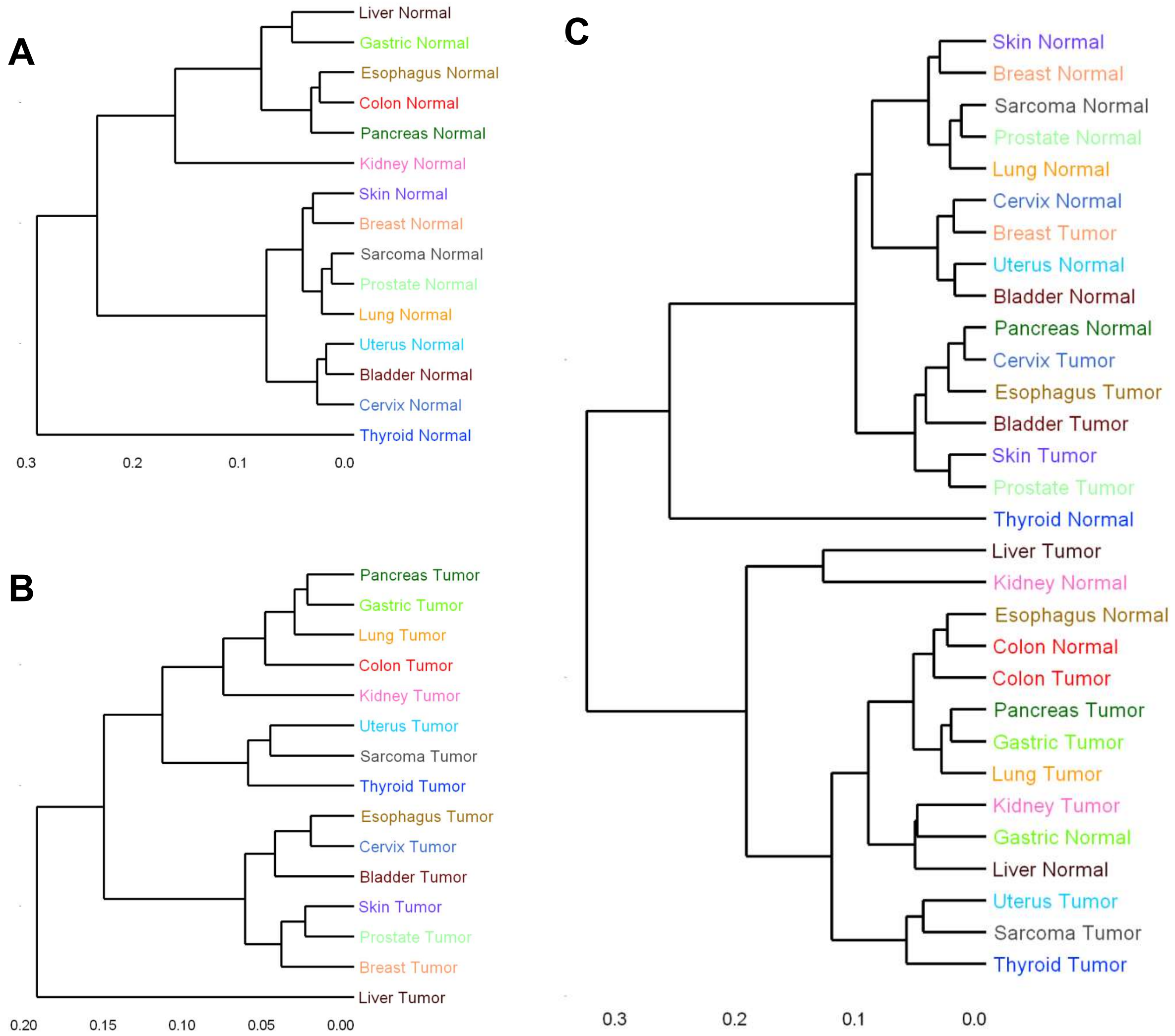

Supplementary Fig. 2. Clustering of glycan averages across tissues. Clustering analysis was performed in R using the Euclidean distances to form a linkage matrix. **A.** Normal tissue clustering. **B.** Tumor tissue clustering. **C.** Clustering of all tissues both normal and tumor.

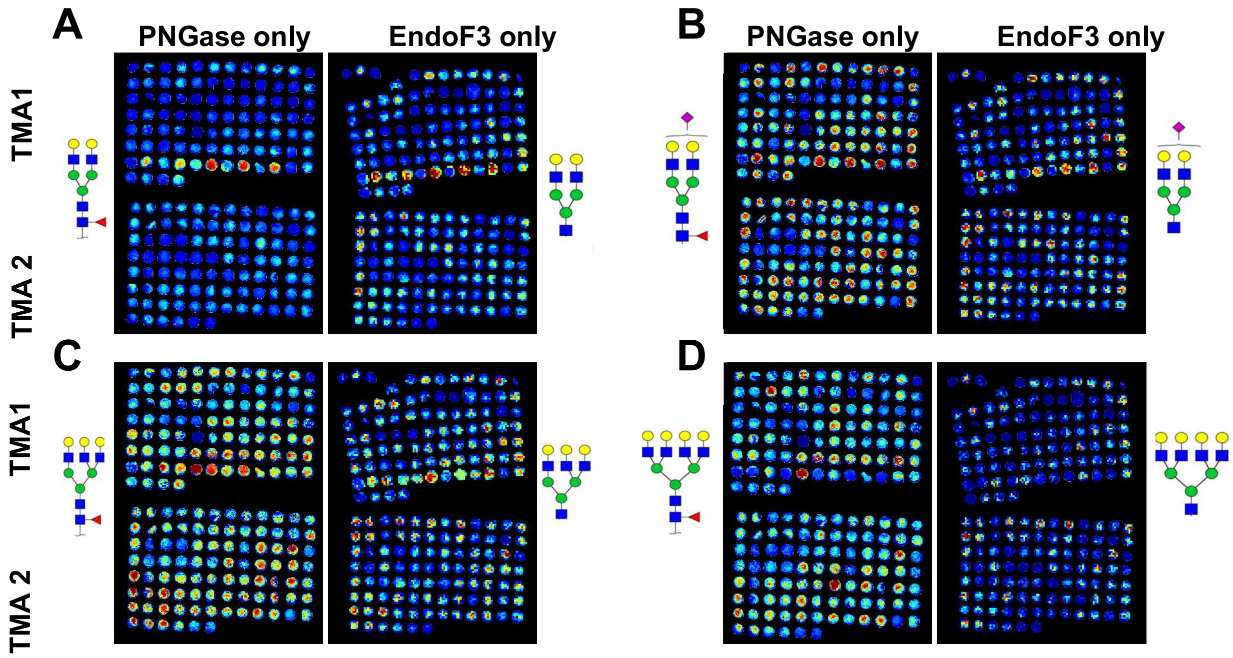

Supplementary Fig. 3. EndoF3 shows core vs. outer-arm fucosylation of *N*-glycans. Comparing PNGaseF digestion (whole glycan) to EndoF3 digestion (truncation above core fucosylation) of the most abundant bi-, tri- and tetra-antennary core-fucosylated *N*-glycan species shows the proportions of these species that have a core fucose rather than an outer arm fucose. **A.** 1809.64 m/z with PNGase; 1640 m/z with EndoF3. **B.** 2122.73 m/z with PNGase; 1773 m/z with EndoF3. **C.** 2174.77 m/z with PNGase; 1825 m/z with EndoF3. **D.** 2539.9037 m/z with PNGase; 2190 m/z with EndoF3.

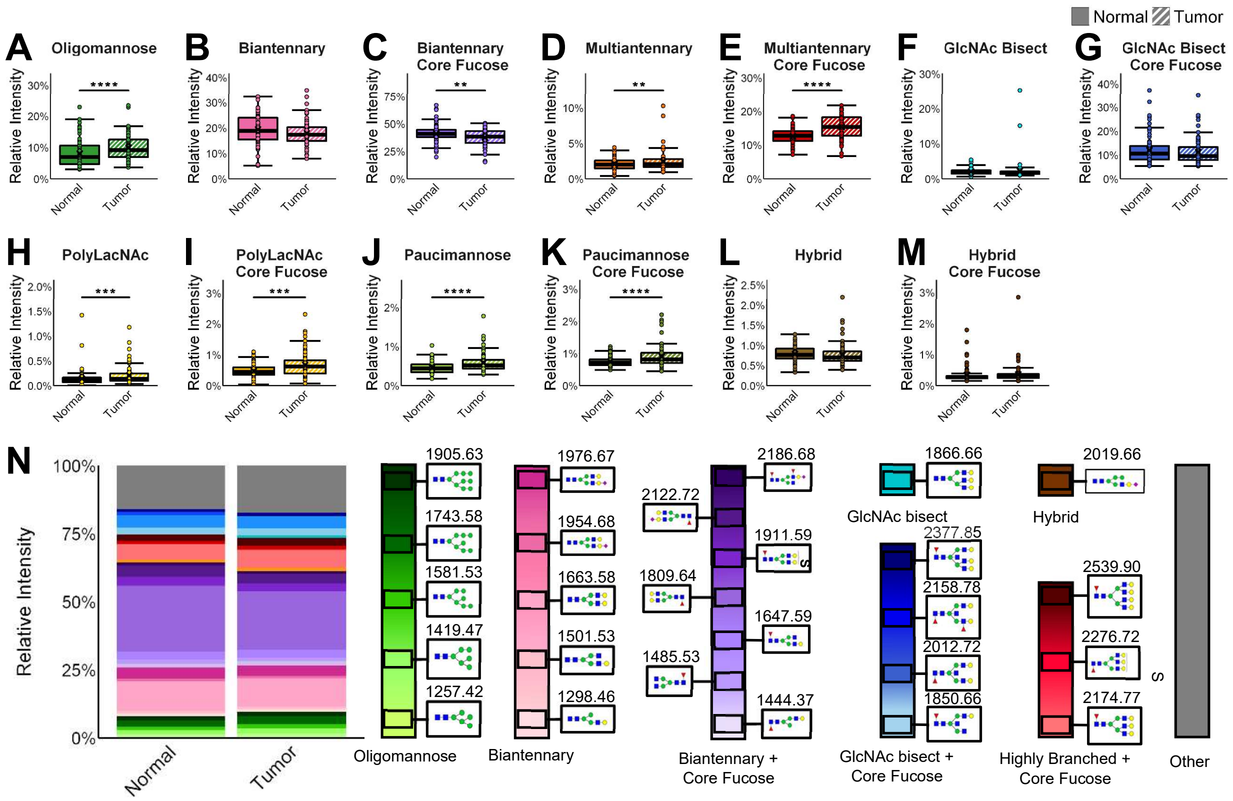

Supplementary Fig. 4. Types and top *N*-glycans for tumor vs. normal across all tissues. (Bladder n=4, Breast n=7, Cervix n=4, Colon n=10, Esophagus n=5, Gastric n=4, Kidney n=3, Liver n=4, Lung n=10, Sarcoma n=4, Skin n=5, Pancreas n=5, Prostate n=8, Thyroid n=6, Uterus n=3) Significance is marked as follows: (*): p-value<0.05; (**): p-value<0.01; (***): p-value<0.001; (****): p-value<0.0001. Error bars on bar charts represent one standard deviation. Error bars on boxplots represent the quartiles. **A.** Oligomannose N-glycans. **B.** Biantennary – no core fucose. **C.** Biantennary – core fucose. **D.** Multiantennary – no core fucose. **E.** Multiantennary – core fucose. **F.** GlcNAc Bisect – no core fucose. **G.** GlcNAc Bisect – core fucose. **H.** PolyLacNAc – no core fucose. **I.** PolyLacNAc – core fucose. **J.** Paucimannose – no core fucose. **K.** Paucimannose – core fucose. **L.** Hybrid N-glycans – no core fucose. **M.** Hybrid N-glycans – core fucose. **N.** Average relative intensities of glycans for total normal and tumor sorted by glycan type. Legend shows glycan structures and glycans are arranged by glycan type in a gradient from smallest m/z to largest.

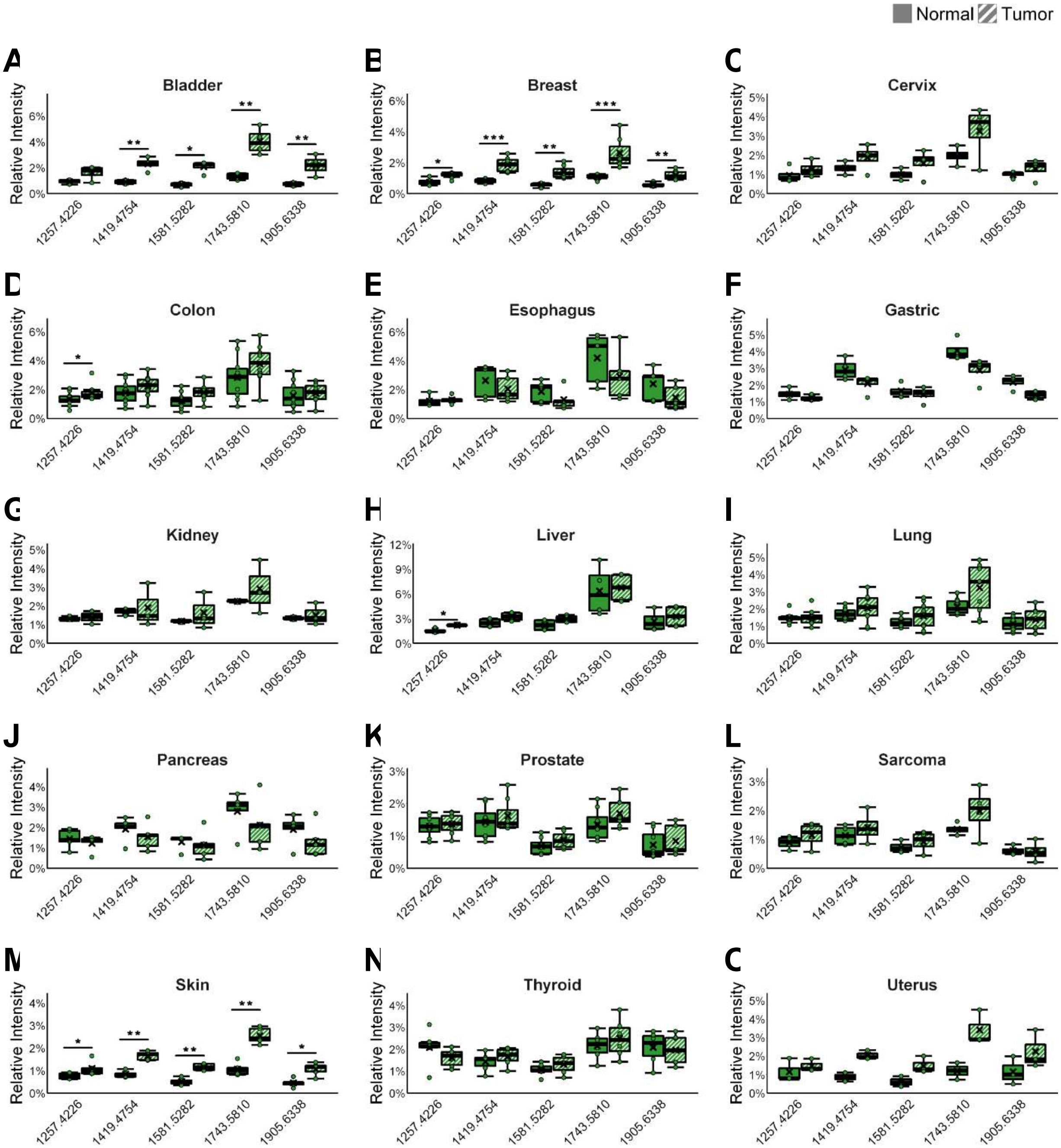

Supplementary Fig. 5. Canonical Oligomannose *N*-glycan expression for all tissue types.

Significance is marked as follows: (*): p-value<0.05; (**): p-value<0.01; (***): p-value<0.001; (****): p-value<0.0001. **A.** Bladder (n=4). **B.** Breast (n=7). **C.** Cervix (n=4). **D.** Colon (n=10). **E.**

Esophagus (n=5). **F.** Gastric (n=4). **G.** Kidney (n=3). **H.** Liver (n=4). **I.** Lung (n=10). **J.** Pancreas (n=5). **K.** Prostate (n=8). **L.** Sarcoma (n=4). **M.** Skin (n=5). **N.** Thyroid (n=6). **O.** Uterus (n=3)

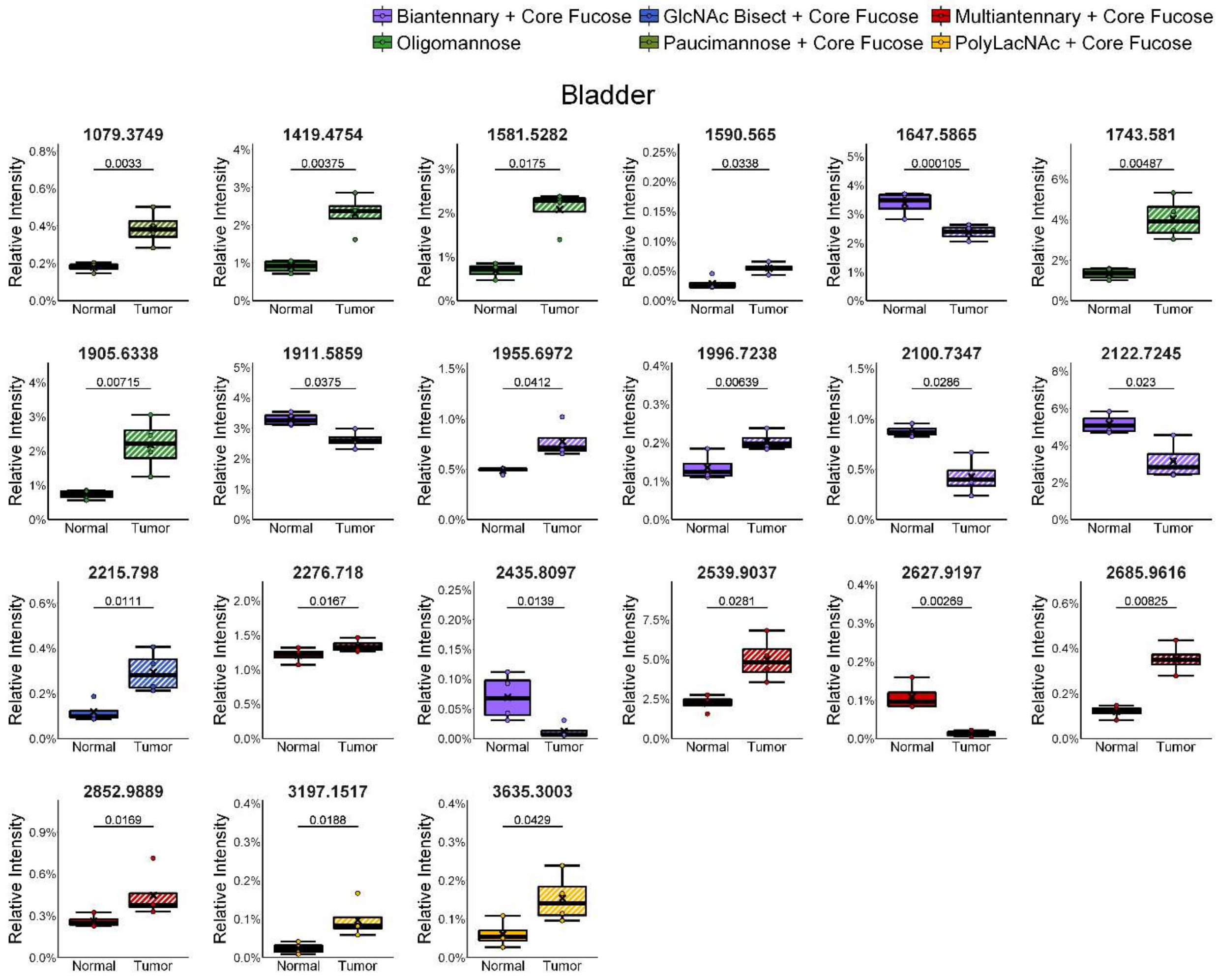

Supplementary Fig. 6. Significant Bladder glycans. (n=4) $p<0.05$, student's paired t-test; relative intensity. Significance is marked as follows: (*): p-value<0.05; (**): p-value<0.01; (***): p-value<0.001; (****): p-value<0.0001. Error bars represent the quartiles.

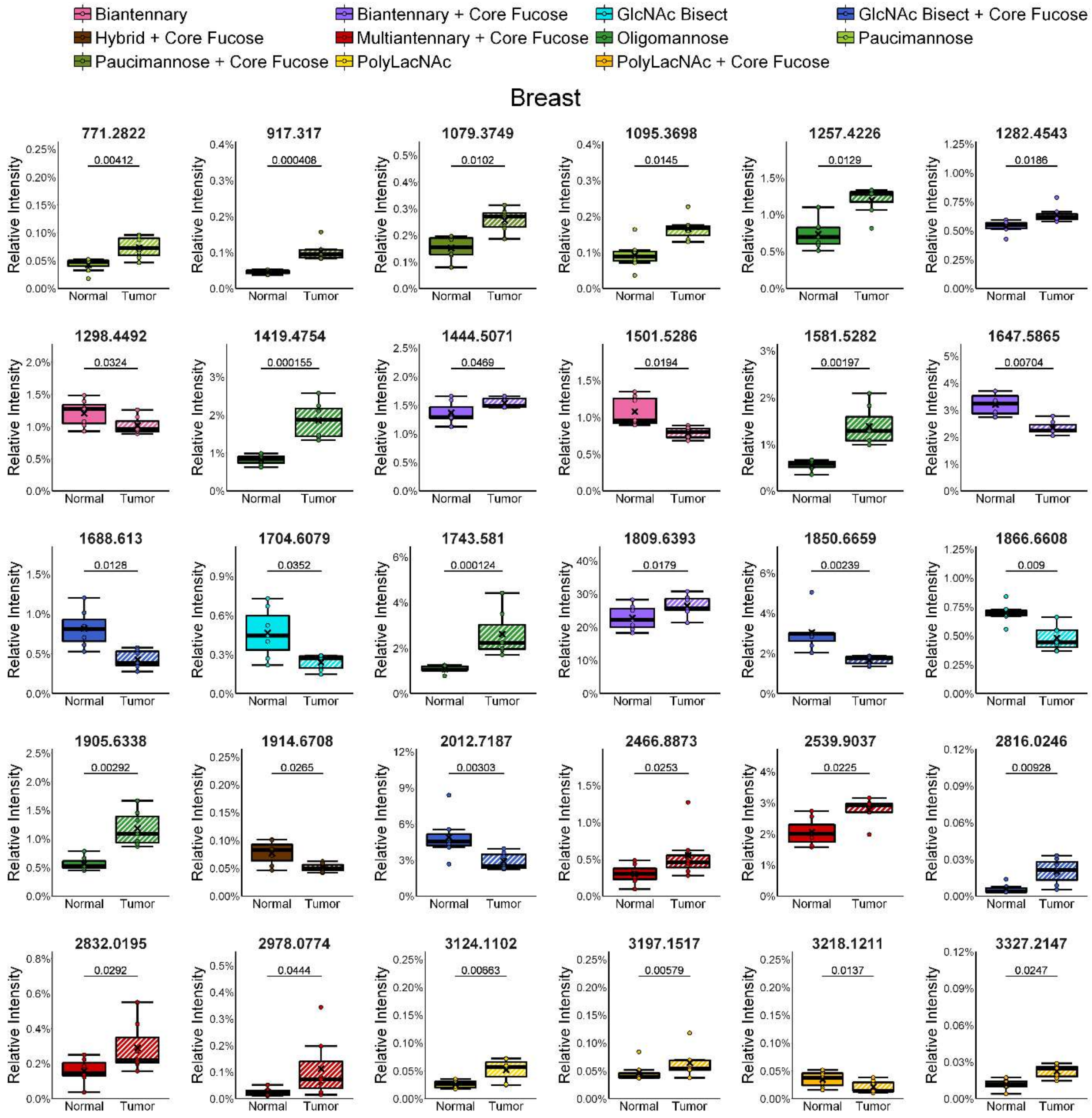

Supplementary Fig. 7. Significant Breast Glycans. (n=7) $p<0.05$, student's paired t-test; relative intensity. Significance is marked as follows: (*): p-value<0.05; (**): p-value<0.01; (***): p-value<0.001; (****): p-value<0.0001. Error bars represent the quartiles.

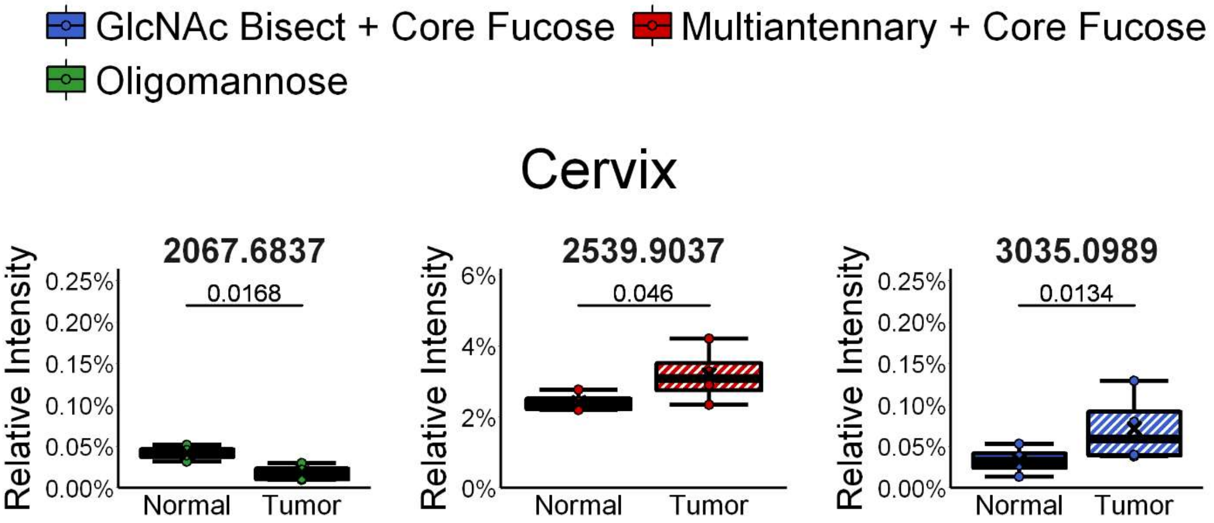

Supplementary Fig. 8. Significant Cervical Glycans. (n=4) $p<0.05$, student's paired t-test; relative intensity. Significance is marked as follows: (*): p-value<0.05; (**): p-value<0.01; (***): p-value<0.001; (****): p-value<0.0001. Error bars represent the quartiles.

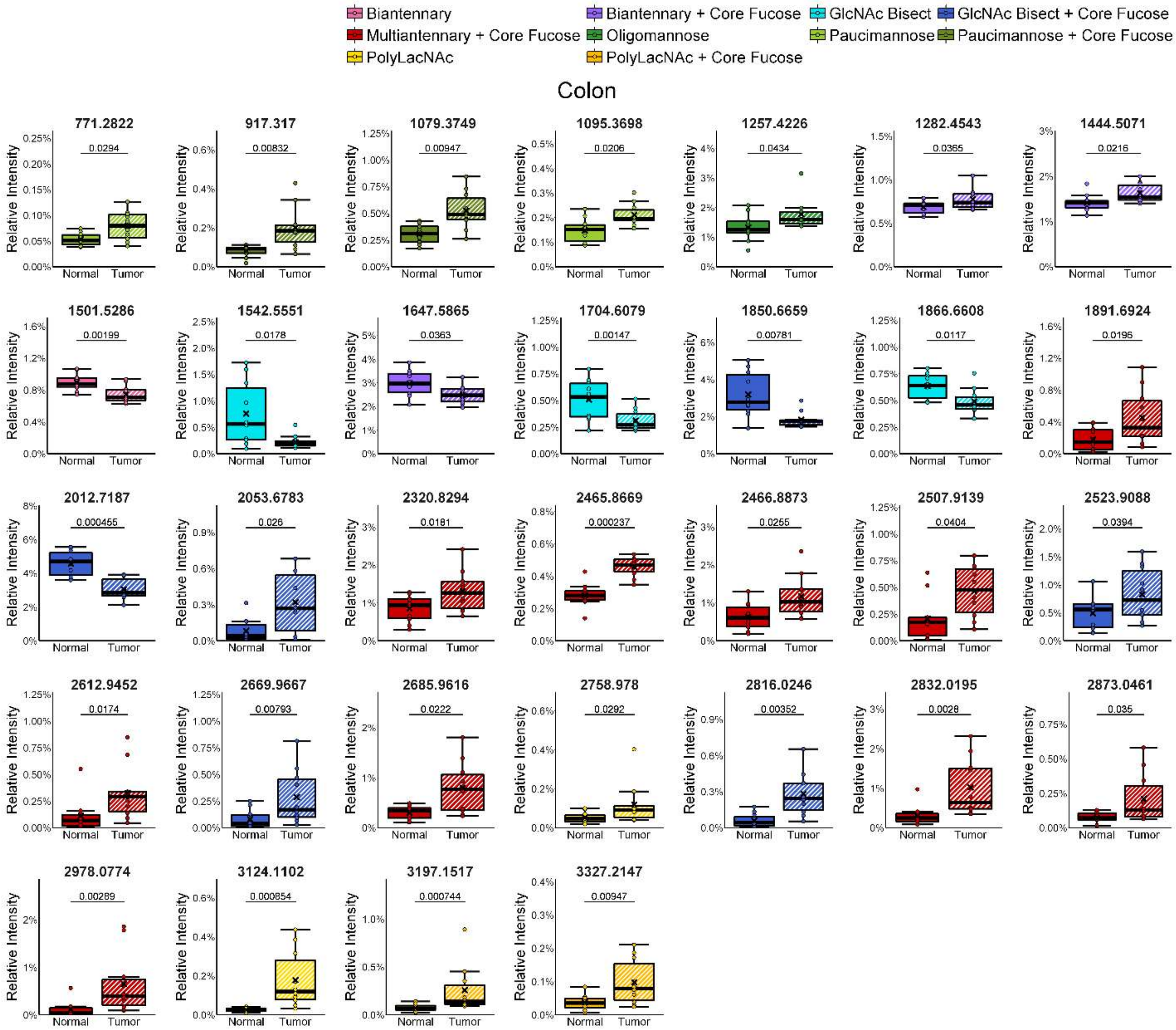

Supplementary Fig. 9. Significant Colon Glycans. (n=10) p<0.05, student's paired t-test; relative intensity. Significance is marked as follows: (*): p-value<0.05; (**): p-value<0.01; (***): p-value<0.001; (****): p-value<0.0001. Error bars represent the quartiles.

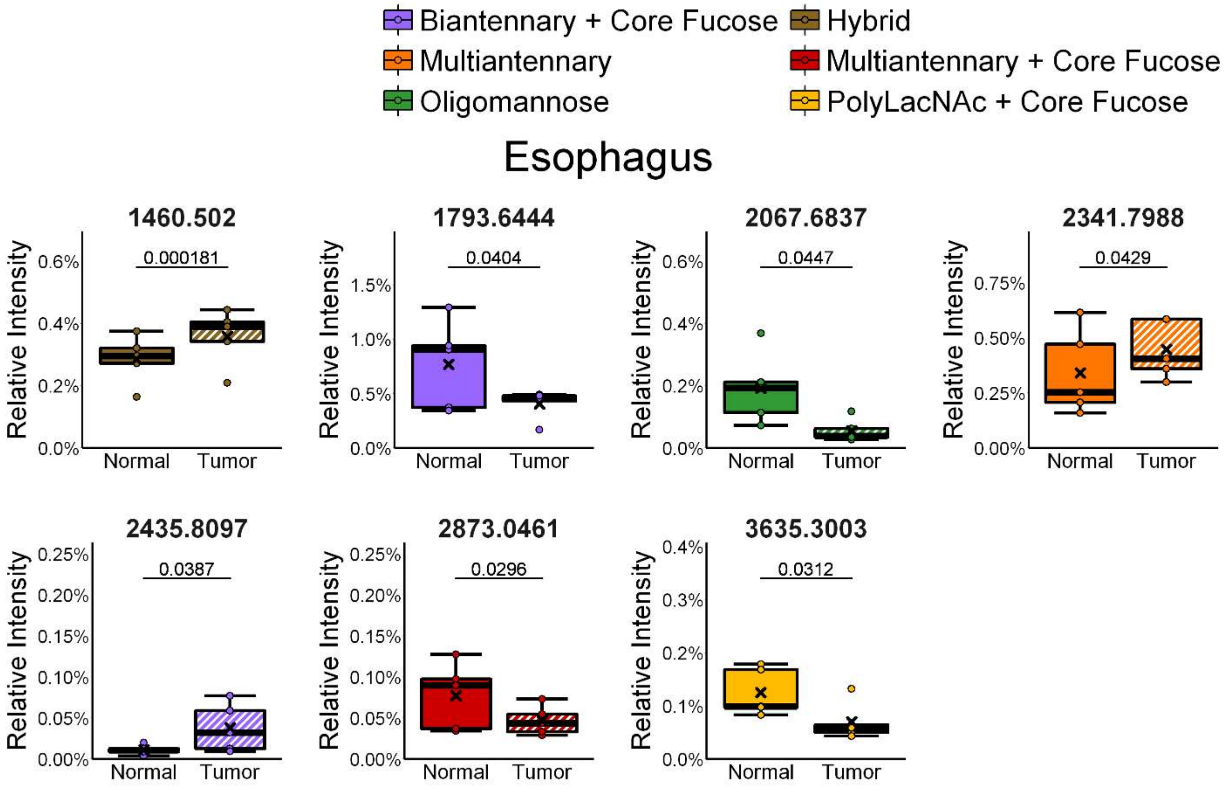

Supplementary Fig. 10. Significant Esophageal Glycans. (n=5) p<0.05, student's paired t-test; relative intensity. Significance is marked as follows: (*): p-value<0.05; (**): p-value<0.01; (***): p-value<0.001; (****): p-value<0.0001. Error bars represent the quartiles.

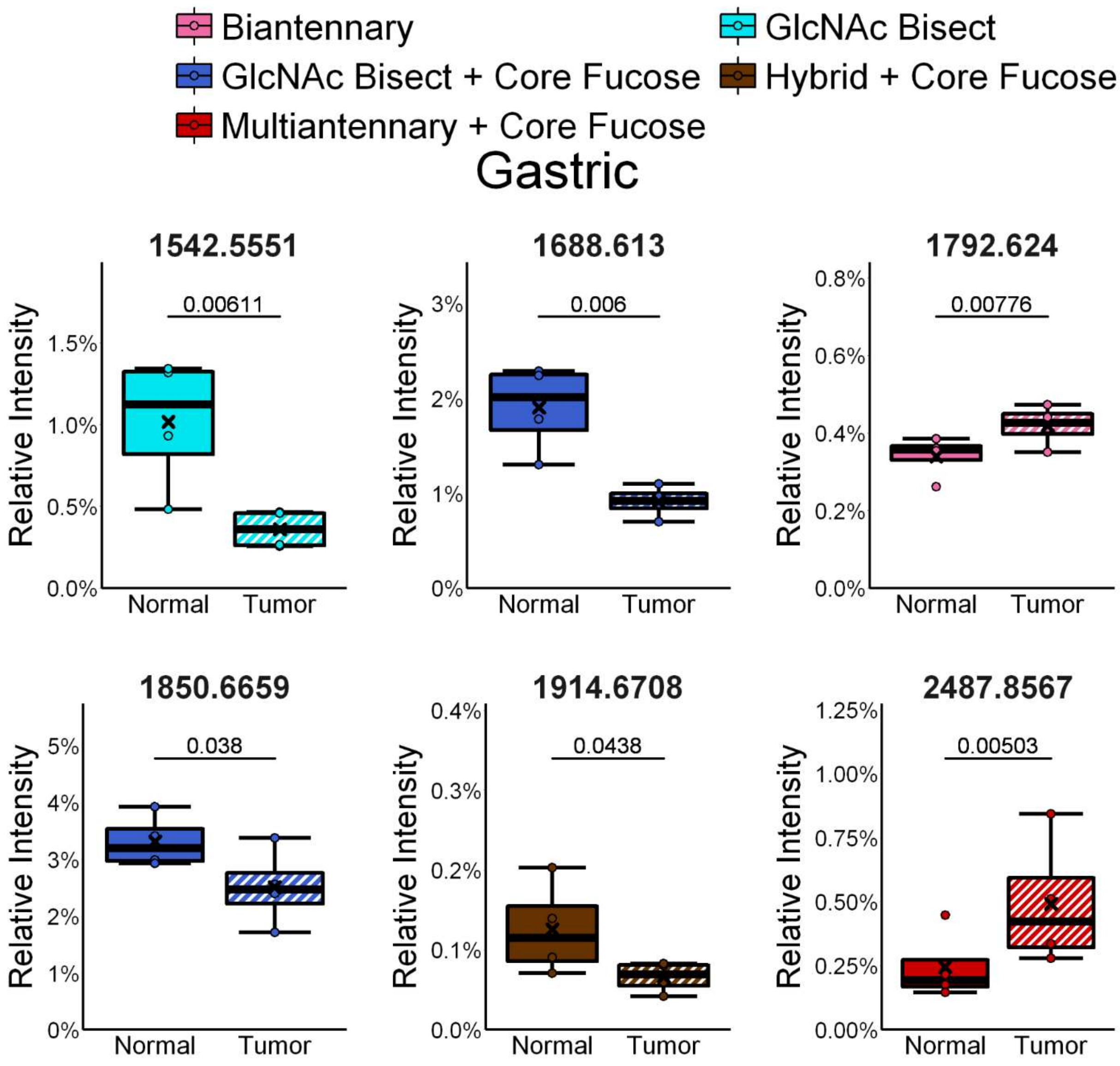

Supplementary Fig. 11. Significant Gastric Glycans. (n=4) $p<0.05$, **student's** paired t-test; relative intensity. Significance is marked as follows: (*): p-value<0.05; (**): p-value<0.01; (***): p-value<0.001; (****): p-value<0.0001. Error bars represent the quartiles.

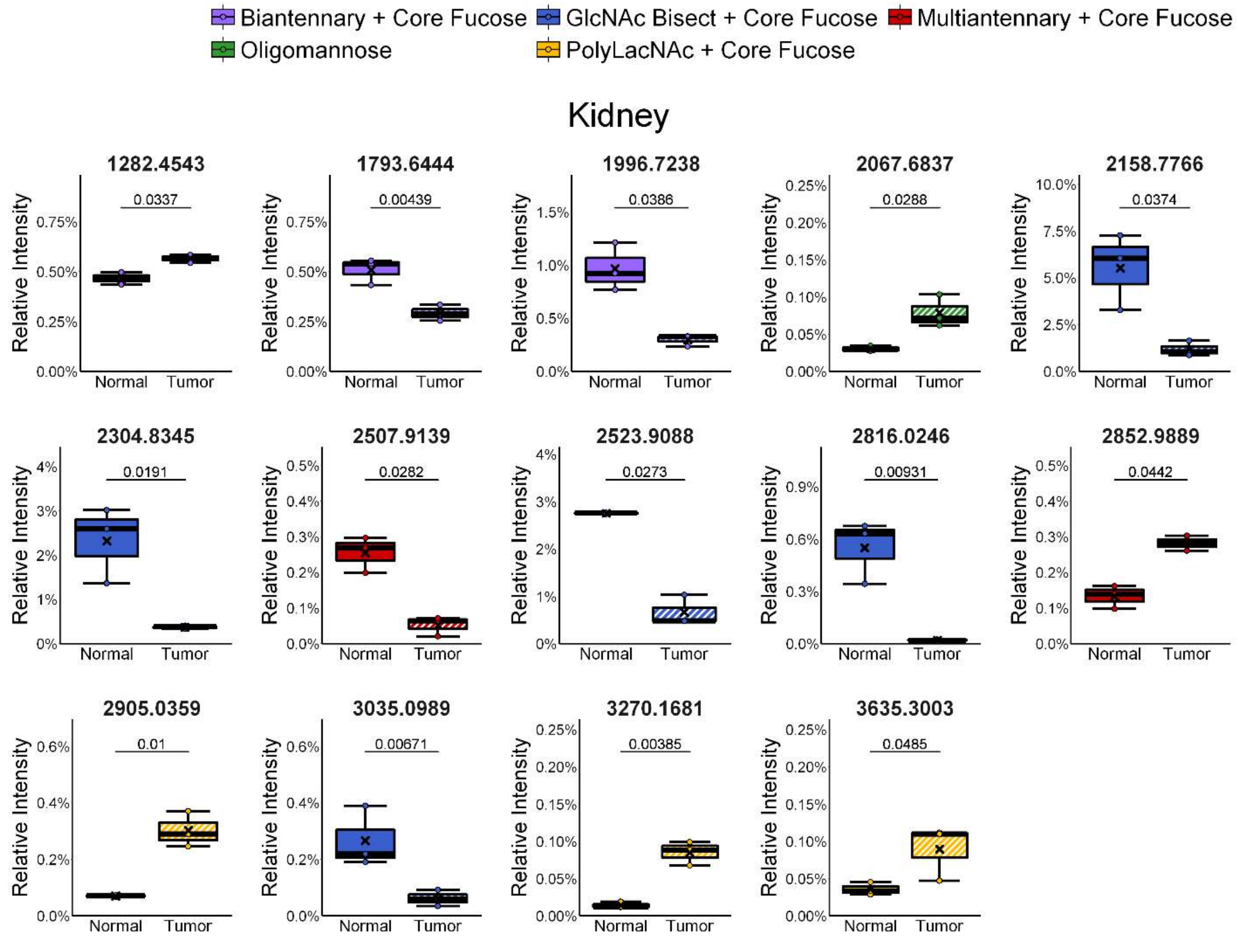

Supplementary Fig. 12. Significant Kidney Glycans. (n=3) $p<0.05$, student's paired t-test; relative intensity. Significance is marked as follows: (*): p-value<0.05; (**): p-value<0.01; (***): p-value<0.001; (****): p-value<0.0001. Error bars represent the quartiles.

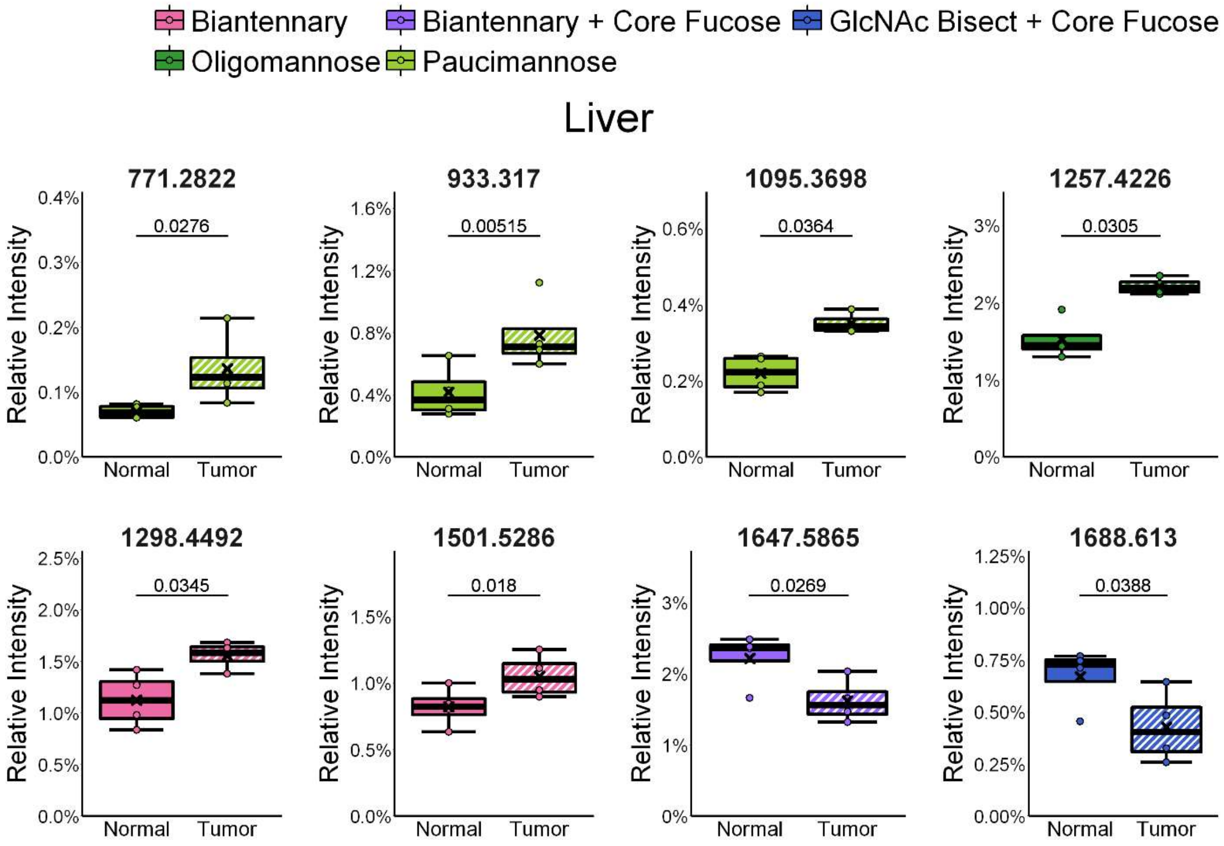

Supplementary Fig. 13. Significant Liver Glycans. (n=4) $p<0.05$, student's paired t-test; relative intensity. Significance is marked as follows: (*): p-value<0.05; (**): p-value<0.01; (***): p-value<0.001; (****): p-value<0.0001. Error bars represent the quartiles.

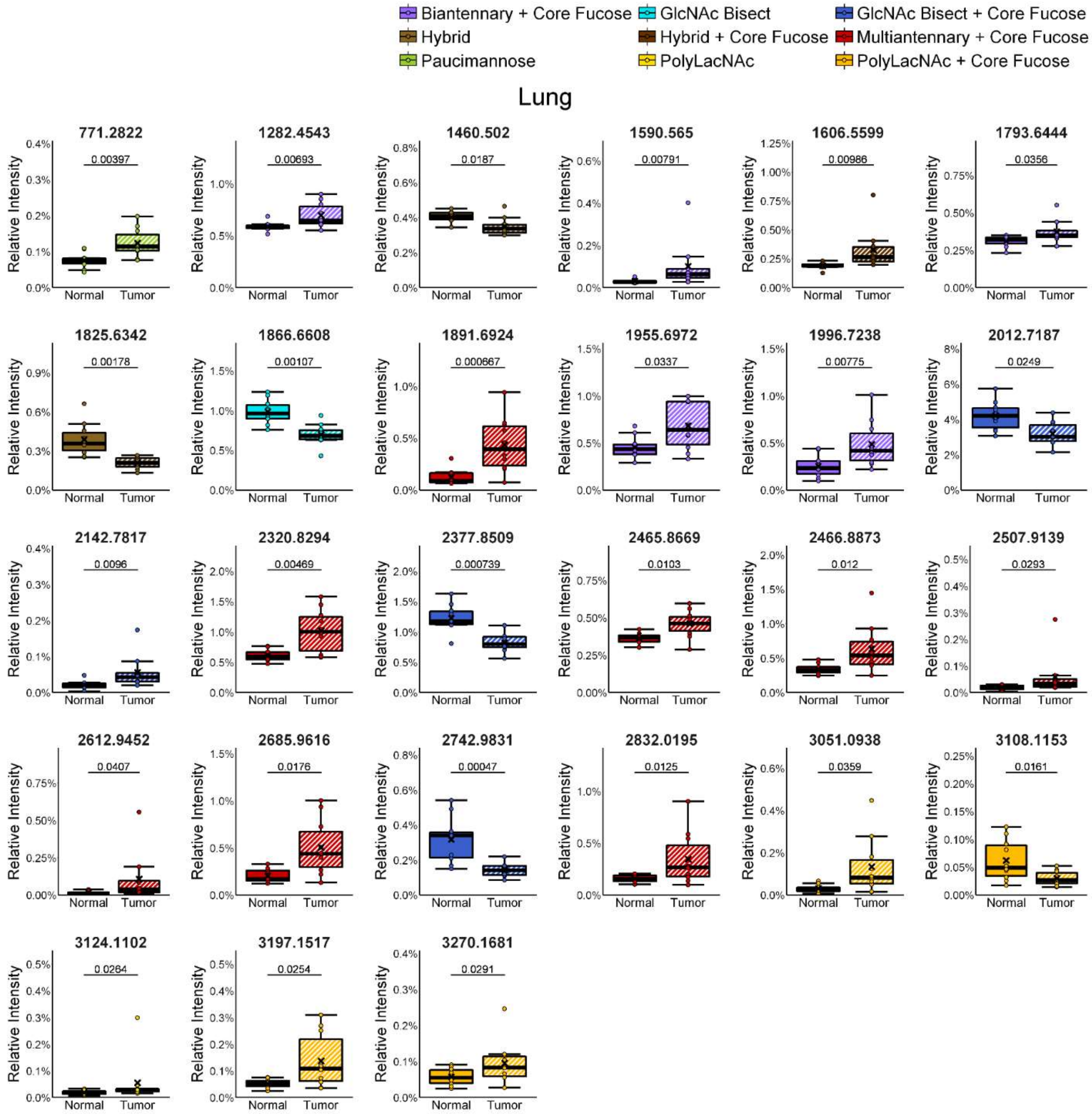

Supplementary Fig. 14. Significant Lung Glycans. (n=10) p<0.05, student's paired t-test; relative intensity. Significance is marked as follows: (*): p-value<0.05; (**): p-value<0.01; (***): p-value<0.001; (****): p-value<0.0001. Error bars represent the quartiles.

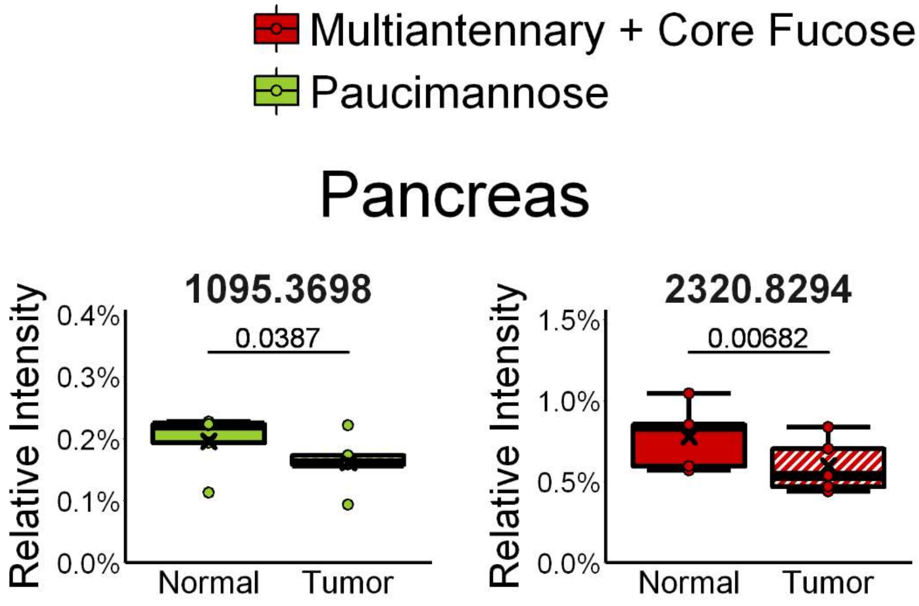

Supplementary Fig. 15. Significant Pancreatic Glycans. (n=5) $p<0.05$, student's paired t-test; relative intensity. Significance is marked as follows: (*): p-value<0.05; (**): p-value<0.01; (***): p-value<0.001; (****): p-value<0.0001. Error bars represent the quartiles.

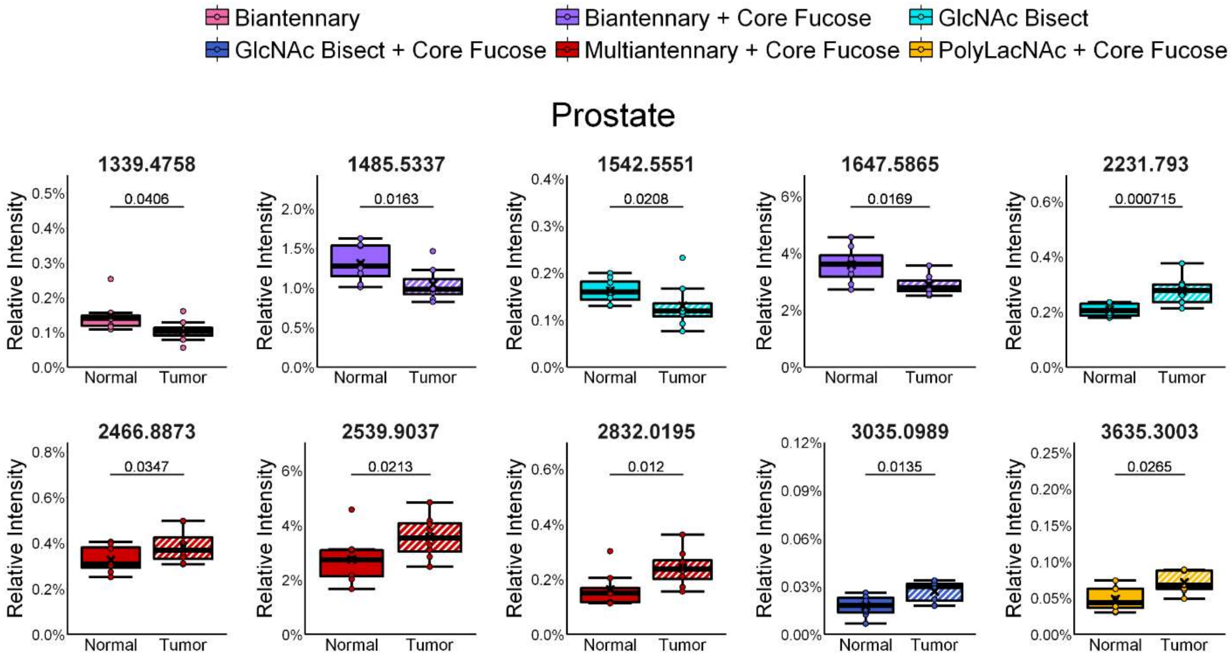

Supplementary Fig. 16. Significant Prostate Glycans. (n=8) p<0.05, student's paired t-test; relative intensity. Significance is marked as follows: (*): p-value<0.05; (**): p-value<0.01; (***): p-value<0.001; (****): p-value<0.0001. Error bars represent the quartiles.

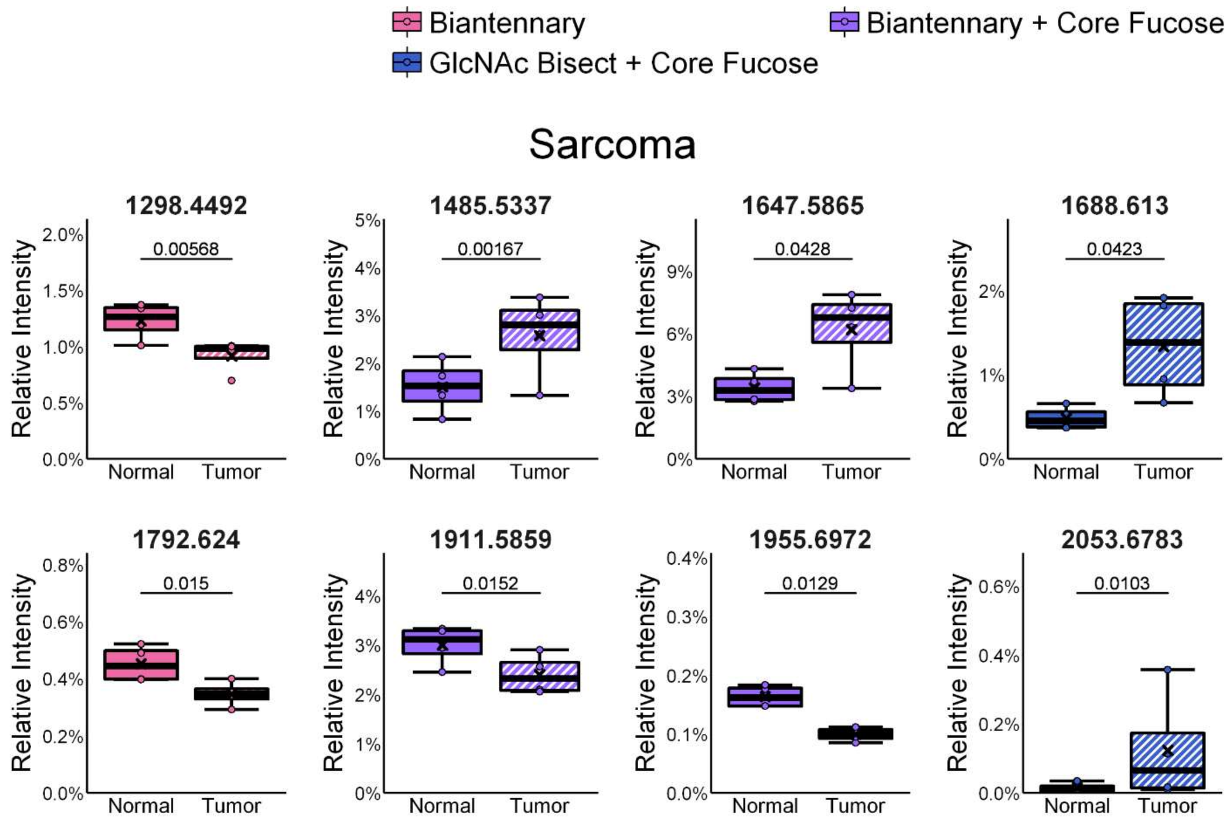

Supplementary Fig. 17. Significant Sarcoma Glycans. (n=4) $p<0.05$, student's paired t-test; relative intensity. Significance is marked as follows: (*): p-value<0.05; (**): p-value<0.01; (***): p-value<0.001; (****): p-value<0.0001. Error bars represent the quartiles.

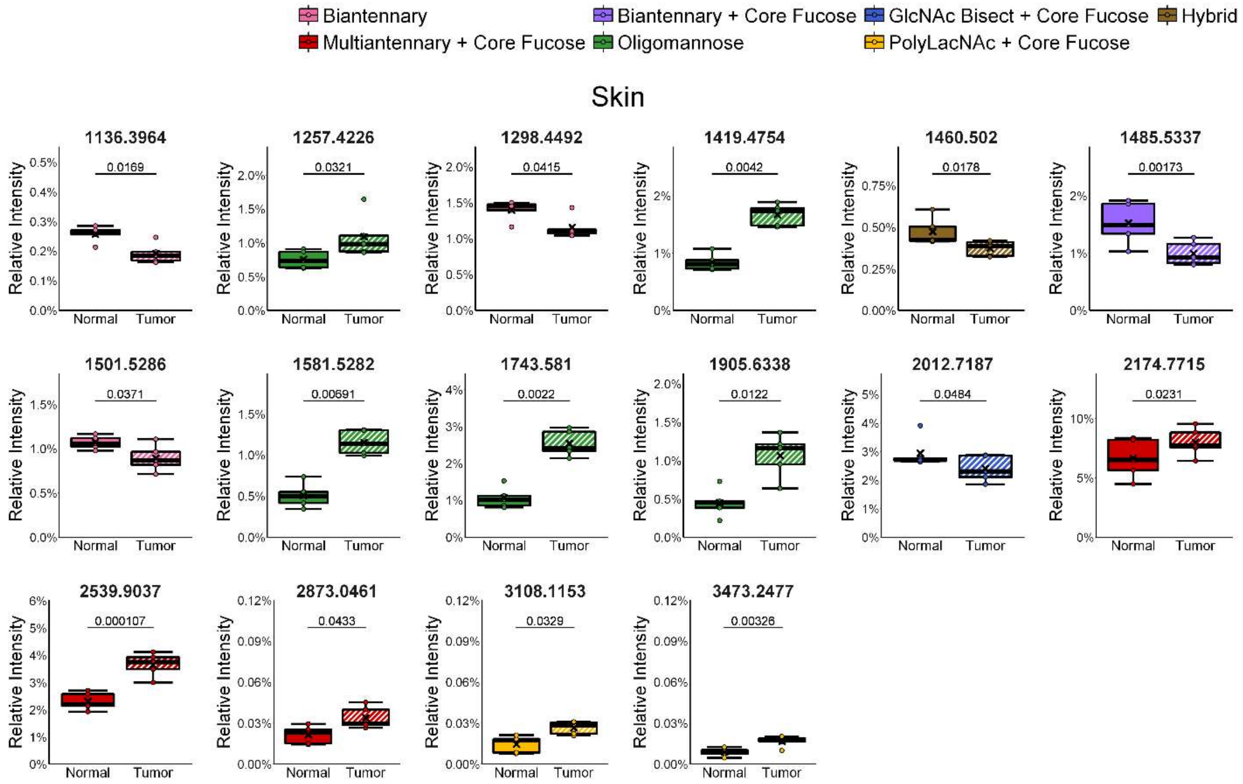

Supplementary Fig. 18. Significant Skin Glycans. (n=5) $p<0.05$, student's paired t-test; relative intensity. Significance is marked as follows: (*): p-value<0.05; (**): p-value<0.01; (***): p-value<0.001; (****): p-value<0.0001. Error bars represent the quartiles.

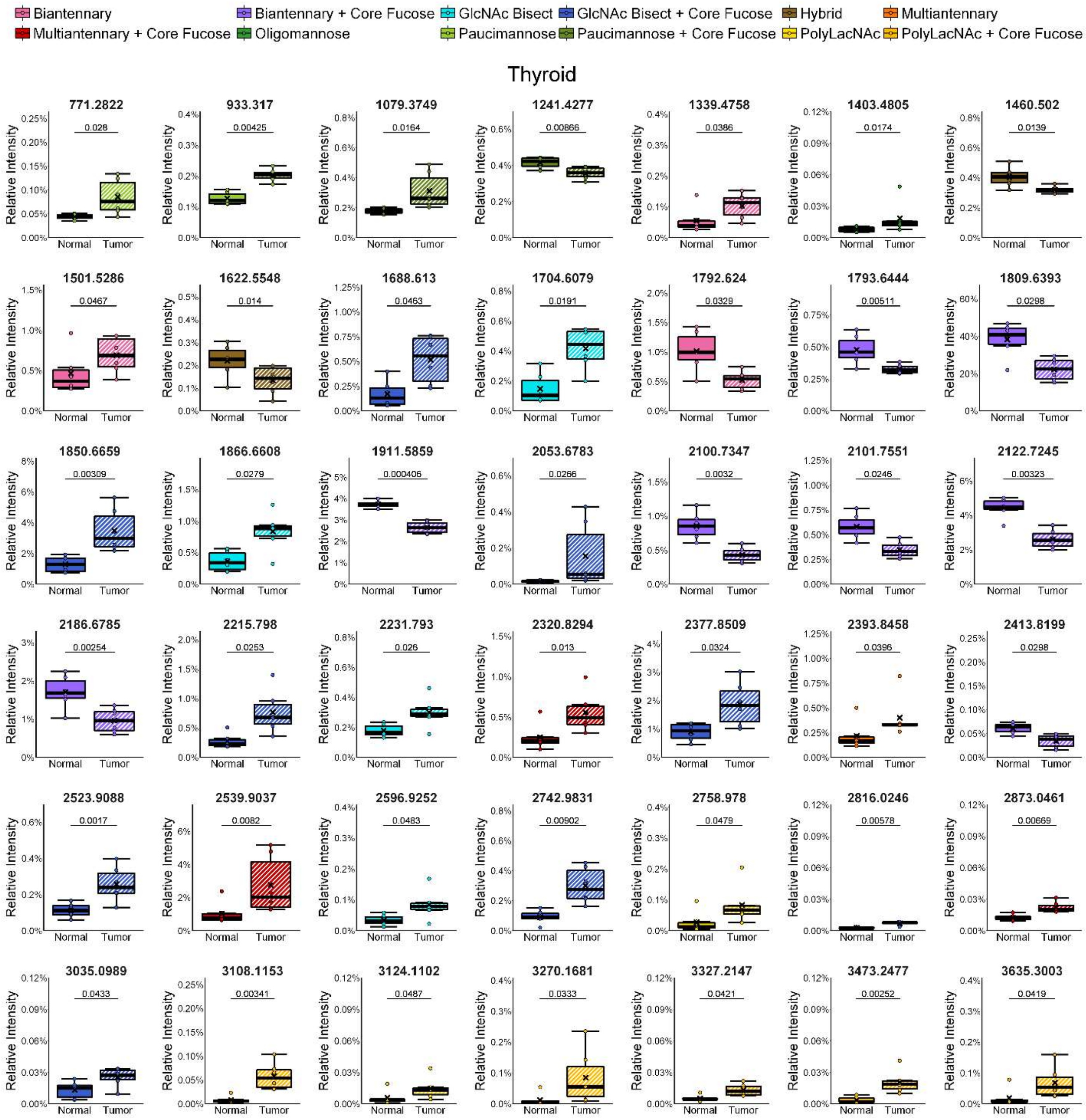

Supplementary Fig. 19. Significant Thyroid Glycans. (n=6) p<0.05, student's paired t-test; relative intensity. Significance is marked as follows: (*): p-value<0.05; (**): p-value<0.01; (***): p-value<0.001; (****): p-value<0.0001. Error bars represent the quartiles.

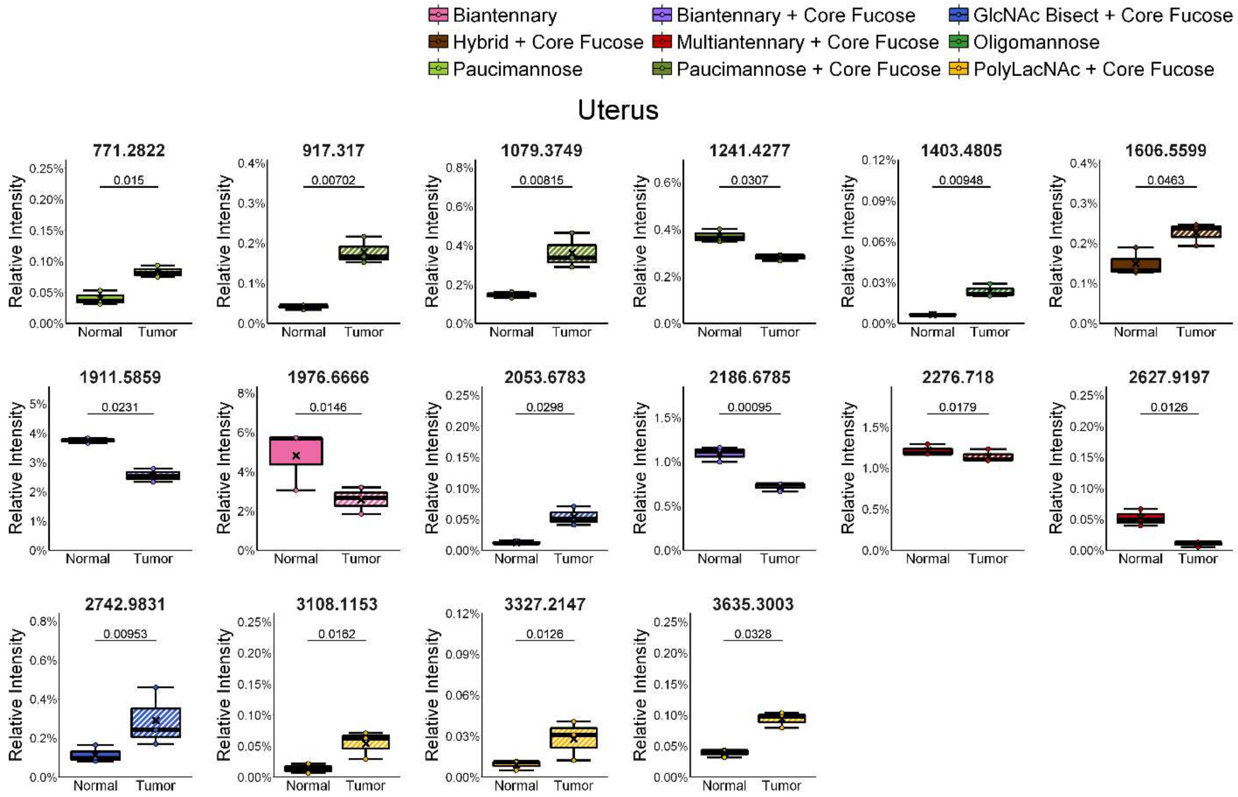

Supplementary Fig. 20. Significant Uterine Glycans. (n=3) $p<0.05$, student's paired t-test; relative intensity. Significance is marked as follows: (*): p-value<0.05; (**): p-value<0.01; (***): p-value<0.001; (****): p-value<0.0001. Error bars represent the quartiles.

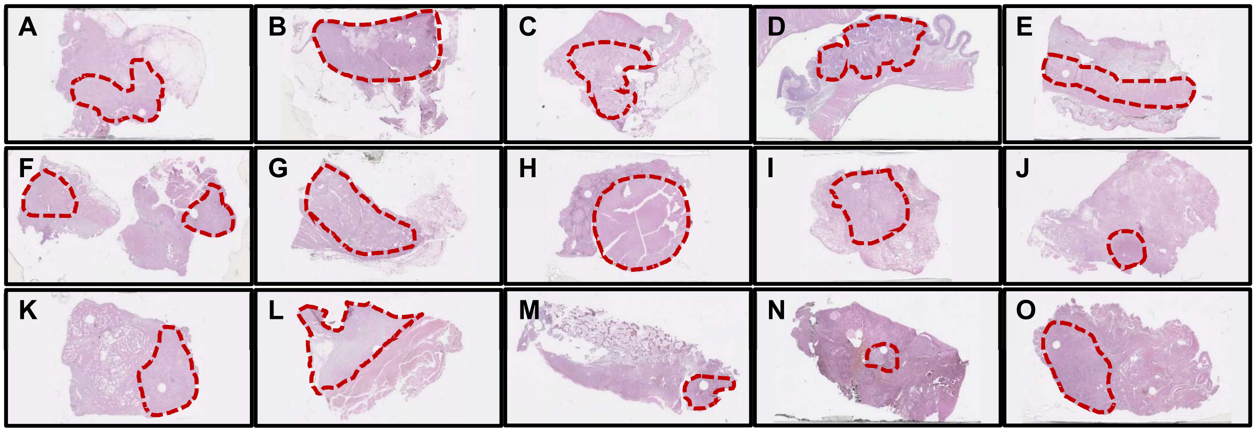

Supplementary Fig. 21. Large tissue H&E stains. Cancerous regions are outlined in red. **A.** Bladder; **B.** Breast; **C.** Cervix; **D.** Colon; **E.** Esophagus; **F.** Gastric; **G.** Kidney; **H.** Liver; **I.** Lung; **J.** Pancreas; **K.** Prostate; **L.** Sarcoma; **M.** Skin; **N.** Thyroid; **O.** Uterus.
