# Supplementary material for: An N-glycome tissue atlas of 15 human normal and cancer tissue types determined by MALDI-imaging mass spectrometry
Source: Sci Rep. 2024 Jan 4;14:489. doi: 10.1038/s41598-023-50957-w (PMC10766640; doi:10.1038/s41598-023-50957-w)
Supplement: Supplementary file 2 — Supplementary Legends. [file 41598_2023_50957_MOESM2_ESM.docx]

**Supporting Information**

**S1 Fig. Top 20 global glycans.** *N-*glycans comprising the top 20 glycans when all tissue data was summed, ranked by abundance.

**S2 Fig. Clustering of glycan averages across tissues.** Clustering analysis was performed in R using the Euclidean distances to form a linkage matrix. (A) Normal tissue clustering. (B) Tumor tissue clustering. (C) Clustering of all tissues both normal and tumor.

**S3 Fig. EndoF3 shows core vs. outer-arm fucosylation of *N-*glycans.** Comparing PNGaseF digestion (whole glycan) to EndoF3 digestion (truncation above core fucosylation) of the most abundant bi-, tri- and tetra-antennary core-fucosylated *N-*glycan species shows the proportions of these species that have a core fucose rather than an outer arm fucose. (A) 1809.64 m/z base glycan. (B) 2122.73 m/z base glycan. (C) 2174.77 m/z base glycan. (D) 2539.9037 m/z base glycan.

**S4 Fig.** A-G Glycans graphed by glycan category. Significant glycans (student’s paired t-test) are marked as follows: (*): p-value<0.05; (**): p-value<0.02; (***): p-value<0.001. (A) Paucimannose glycans: *N-*glycans containing only mannose residues on the branches with less than 5 mannose residues; (B) Mannose-only glycans: *N-*glycans containing only mannose residues on the branches; (C) Hybrid glycans: *N-*glycans containing a mannose branch and a mixed-residue branch; (D) Biantennary: glycans with two branches of mixed residues; (E) Multiantennary: glycans with three or four branches of mixed residues; (F) GlcNAc Bisect: glycans with an *N-*acetyl glucosamine bisect; (G) PolyLacNAc: glycans with a branch ending in a repeating chain of galactose and *N-*Acetyl glucosamine dimers; (H) Average relative intensities of glycans for total normal and tumor sorted by glycan type. Legend shows glycan structures and glycans are arranged by glycan type in a gradient from smallest m/z to largest.

**S5 Fig. Canonical High Mannose *N-*glycan expression for all tissue types.**

**S6 Fig. Significant Bladder glycans.** (n=4) p<0.05, student’s paired t-test; relative intensity. Error bars represent the quartiles.

**S7 Fig. Significant Breast Glycans.** (n=7) p<0.05, student’s paired t-test; relative intensity. Error bars represent the quartiles.

**S8 Fig. Significant Cervical Glycans.** (n=4) p<0.05, student’s paired t-test; relative intensity. Error bars represent the quartiles.

**S9 Fig. Significant Colon Glycans.** (n=10) p<0.05, student’s paired t-test; relative intensity. Error bars represent the quartiles.

**S10 Fig. Significant Esophageal Glycans.** (n=5) p<0.05, student’s paired t-test; relative intensity. Error bars represent the quartiles.

**S11 Fig. Significant Gastric Glycans.** (n=4) p<0.05, **student’s** paired t-test; relative intensity. Error bars represent the quartiles.

**S12 Fig. Significant Kidney Glycans.** (n=3) p<0.05, student’s paired t-test; relative intensity. Error bars represent the quartiles.

**S13 Fig. Significant Liver Glycans.** (n=4) p<0.05, student’s paired t-test; relative intensity. Error bars represent the quartiles.

**S14 Fig. Significant Lung Glycans.** (n=10) p<0.05, student’s paired t-test; relative intensity. Error bars represent the quartiles.

**S15 Fig. Significant Pancreatic Glycans.** (n=5) p<0.05, student’s paired t-test; relative intensity. Error bars represent the quartiles.

**S16 Fig. Significant Prostate Glycans.** (n=8) p<0.05, student’s paired t-test; relative intensity. Error bars represent the quartiles.

**S17 Fig. Significant Sarcoma Glycans.** (n=4) p<0.05, student’s paired t-test; relative intensity. Error bars represent the quartiles.

**S18 Fig. Significant Skin Glycans.** (n=5) p<0.05, student’s paired t-test; relative intensity. Error bars represent the quartiles.

**S19 Fig. Significant Thyroid Glycans.** (n=6) p<0.05, student’s paired t-test; relative intensity. Error bars represent the quartiles.

**S20 Fig. Significant Uterine Glycans.** (n=3) p<0.05, student’s paired t-test; relative intensity. Error bars represent the quartiles.

**S21 Fig. Large tissue H&E stains.** Cancerous regions are outlined in red. (A) Bladder (B) Breast (C) Cervix (D) Colon (E) Esophagus (F) Gastric (G) Kidney (H) Liver (I) Lung (J) Pancreas (K) Prostate (L) Sarcoma (M) Skin (N) Thyroid (O) Uterus.

**S1 Table. TMA tissues and pathologist annotations.**

**S2 Table. *N-*glycans detected with PNGase.**

**S3 Table. Sialylated *N-*glycans detected with AAXL and PNGase.**

**S1 File. Raw and Relative Intensity data from PNGase treatment.**

**S2 File. Raw and Relative Intensity data from EndoF3 treatment.**

**S3 File. Raw and Relative Intensity data from AAXL and PNGase treatment.**
